# Factor Structure and Measurement Invariance Across Gender of the Eating Disorder Examination Questionnaire—Short Form in Italian Workers

**DOI:** 10.3390/ejihpe16030037

**Published:** 2026-03-05

**Authors:** Nicola Magnavita, Carlo Chiorri

**Affiliations:** 1 Department of Safety and Bioethics, Università Cattolica del Sacro Cuore, 00168 Roma, Italy; 2 Department of Educational Sciences, University of Genova, 16128 Genova, Italy

**Keywords:** psychometry, graded response model, measurement invariance, gender differences, workplace, health promotion, health surveillance, screening, prevention, mental health, metabolic disorders

## Abstract

Eating disorders (EDs) are complex conditions that can significantly affect health and productivity, yet their assessment in occupational settings remains underexplored. This study aimed to evaluate the psychometric properties of the Italian version of the Eating Disorder Examination Questionnaire—Short Form (EDE-QS) among 1912 workers undergoing health surveillance. Using an Item Response Theory framework, we tested dimensionality, reliability, and measurement invariance across gender, applying a graded response model to assess item discrimination and threshold parameters. Results supported an approximate unidimensional structure with excellent internal consistency (ω ≈ 0.95) and strong indices of factor score determinacy and construct replicability. Measurement invariance analyses indicated configural and metric invariance but not full scalar invariance, due to differential item functioning in a subset of items. Latent mean differences were small, with women scoring slightly higher than men, and associations with psychological, occupational, and health-related variables did not differ by gender. These findings indicate that the Italian EDE-QS shows promising structural validity as a brief measure of ED symptomatology in occupational samples in workplace contexts. However, gender-related item bias warrants cautious interpretation of specific behaviors, suggesting the need for tailored assessments to enhance diagnostic accuracy and inform preventive interventions.

## 1. Introduction

Eating disorders (EDs) are complex disorders with multiple causes, as defined by the Diagnostic and Statistical Manual of Mental Disorders (DSM-5-TR; American Psychiatric Association, 2022) and the World Health Organization (WHO) International Classification of Diseases and Related Health Problems (ICD-11; World Health Organization, 2019). They are a heterogeneous set of persistent alterations in eating behavior accompanied by an intense emotional component (Feng et al., 2023). The most common forms include anorexia nervosa (AN), bulimia nervosa (BN), binge eating disorder (BED), and other feeding and ED (Treasure et al., 2020). EDs tend to be more frequent in adolescence and early adulthood (Silén & Keski-Rahkonen, 2022; Smink et al., 2012) but in more than 25% of cases are persistent or recurrent and are still present after more than 5 years of follow-up (Solmi et al., 2024). Moreover, these disorders can also appear in adulthood (Davies, 2024; Samuels et al., 2019; Taba et al., 2021, p. 20; Ward et al., 2019). Clusters of first-onset cases may be associated with a concomitant medical condition (e.g., during the perimenopausal phase (J. H. Baker & Runfola, 2016); following cancer treatment (Rakusin et al., 2021); in polycystic ovary syndrome (Thannickal et al., 2020) or with psychological factors such as perfectionism, emotional discomfort, maladaptive coping mechanisms, insecure attachment patterns developed in childhood, emotional dysregulation, and mood disorders (for reviews, see Barakat et al., 2023; Holland et al., 2013; Solmi et al., 2021; Striegel-Moore & Bulik, 2007; Varela et al., 2023; Zanella & Lee, 2022).

EDs are challenging to treat (De Boer et al., 2023; Johnson & Taylor, 1996; Touma et al., 2023) and have a relatively high mortality rate (Amiri & Ab Khan, 2025; Arcelus et al., 2011; Krug et al., 2025). Moreover, they often go undetected (Baranauskas et al., 2022; Cachelin & Striegel-Moore, 2006). It has been estimated that only around one-third of cases are identified by medical professionals and promptly treated (Keski-Rahkonen & Mustelin, 2016). For this reason, a routine assessment of ED symptomatology is a recommended measure in populations exposed to risk factors (Murray et al., 2025).

Work environments represent one of the settings in which these issues should be investigated, given that among ED-determining factors, there are several stressors that can be linked to work, such as night work (Duong et al., 2020; Sørensen et al., 2013), circadian disruption and sleep deprivation (Mendoza, 2019), atypical light exposure (Engin, 2024), night eating (Abbott et al., 2018; Chellappa et al., 2025; Lent et al., 2022), interaction of shift work with chronotype (Amicis et al., 2023; Romo-Nava et al., 2020, 2022), alterations in biological rhythms (Hemmer et al., 2021; Kosmadopoulos et al., 2020; Meléndez-Fernández et al., 2023), disordered sleep (Mason et al., 2019; Nagata et al., 2021; Wilcox et al., 2024), emotional traumas (Convertino et al., 2022; Rienecke et al., 2022; Thomeczek et al., 2023), workplace or school violence and bullying (Day et al., 2022; Magnavita & Isolani, 2025), and occupational stress (King et al., 2009; Medisauskaite & Kamau, 2019; Qi & Wu, 2025).

The frequent association of EDs with metabolic (Dean et al., 2024; Hudson et al., 2010, 2020; Yu & Muehleman, 2023), cardiovascular (Sachs et al., 2016; TODAY Study Group et al., 2022), and mental illnesses (Alcaraz-Ibáñez et al., 2023; Conti et al., 2021; Dolan et al., 2022; Jelinek et al., 2018; Mitchell et al., 2021; Nelson et al., 2022; Rijkers et al., 2019) means that these disorders have a direct impact on productivity (e.g., absenteeism or presenteeism; Ubhi et al., 2025) and high healthcare costs (Ahmed et al., 2025). This should prompt corporate health and safety departments to actively investigate the presence of EDs and initiate policies to promote healthy eating. ED symptomatology assessment can be conducted by occupational physicians, and this is an essential step in improving worker health.

Although the incidence and prevalence of EDs in workplaces are significant, there are few studies conducted on workers. They have mostly focused on young people, such as students (Alhaj et al., 2022), especially medical students (Fekih-Romdhane et al., 2022; Jahrami et al., 2019), or on those with a particular focus on their body, such as athletes (Bratland-Sanda & Sundgot-Borgen, 2013; Chapa et al., 2022; Milano et al., 2020; Neglia, 2021). The presence of EDs has also been frequently studied in military personnel, especially in relation to obesity (Bartlett & Mitchell, 2015; Himmerich et al., 2024; Touma et al., 2023). Further research is needed to determine the prevalence of suspected EDs in workplace settings and to inform strategies that promote nutritional health and facilitate effective treatment.

The assessment tools to be used in workplaces must be simple, quick-to-complete, and easily readable, as the time spent on these activities must not significantly impact productive activities. The occupational physician will be able to thoroughly examine the cases identified by the assessment and, if necessary, refer them to further specialized investigations.

The Eating Disorder Examination (EDE; Cooper et al., 1989; Fairburn & Cooper, 1993) is widely regarded as the gold standard for assessing ED symptomatology. Its self-report counterpart, the Eating Disorder Examination Questionnaire (EDE-Q; Fairburn & Beglin, 1994) has become a prevalent tool for ED assessment and monitoring (e.g., National Institute for Mental Health in England, 2008). However, the replicability of the EDE-Q’s original four-factor structure (restraint, eating concerns, weight concerns, and shape concerns) has been repeatedly questioned. Empirical evidence for this structure is inconsistent, with numerous studies failing to support the model and instead proposing alternative configurations, such as three- or two-factor solutions (Berg et al., 2012; Jenkins & Rienecke, 2022) Even large, heterogeneous samples have produced results that diverge substantially from the original framework (Burke et al., 2017; Kjeldbjerg et al., 2021). These issues are particularly pronounced in cross-cultural research, where symptom expression may vary, prompting calls for structural modifications to enhance validity across diverse populations (Costello et al., 2023; Heiss et al., 2018; Lichtenstein et al., 2021).

The original EDE-Q (28 items, 28-day reference) is unsuitable for session-by-session monitoring, as it cannot reliably capture weekly changes (Gideon et al., 2016). To address this limitation, several abbreviated versions have been proposed (for a review, see Machado et al., 2020). Gideon et al. (2016) developed the EDE-QS, a 12-item short form derived through statistical analyses and expert review. Modifications included reducing the response scale from six to four frequency levels and shortening the recall period to seven days, enhancing recall accuracy and sensitivity to short-term change. Initial validation suggested adequate internal consistency and temporal stability in adults, supporting its use for routine clinical outcome monitoring. Subsequent studies supported the EDE-QS’s reliability and validity across culturally diverse populations (Duffy et al., 2021; He et al., 2021; Mousavi Asl et al., 2021; Prnjak et al., 2020).

Cross-cultural adaptations of the EDE-QS (Dahlgren et al., 2025; Duffy et al., 2021; He et al., 2021; Mousavi Asl et al., 2021; Prnjak et al., 2020) have generally supported its validity and reliability across culturally diverse populations, and the unidimensional structure derived from Rasch modeling. However, differential item functioning (DIF) has emerged across gender groups in both Norwegian adolescents (Dahlgren et al., 2025) and Chinese college students (He et al., 2021). Specifically, item 6 (“desire to lose weight”) exhibited substantial DIF in the Chinese sample, while the Norwegian study identified similar DIF for item 6 and an additional effect for item 5 (“fear of weight gain”). These findings suggest that, at equivalent levels of symptom severity, women are more likely than men to endorse stronger weight-loss desires and fear of weight gain.

These findings align with prior evidence on gender differences in ED symptomatology, reflecting the interplay of sociocultural norms, body image ideals, and clinical presentation. The status of knowledge indicates that gender and sex influence ED risk (Breton et al., 2023). This observation, derived from clinical studies, is supported by surveys conducted on workers and military personnel (Peleg et al., 2024; Cary et al., 2025). Findings from a systematic review (Sobieska et al., 2025) suggested that sex/gender is among the predictors of ED symptoms. Research consistently shows that women report higher levels of disordered eating characterized by shape and weight concerns, whereas men more often exhibit symptoms related to muscularity and body composition (Lavender et al., 2017; Schaefer et al., 2018, 2019). For example, Schaefer et al. (2018, 2019) noted that EDE-Q items may better capture female symptom profiles. Additionally, men appear less likely to identify with conventional diagnostic labels, contributing to underreporting of disordered eating behaviors (Kinasz et al., 2016; Lavender et al., 2017). Overall, while core symptoms overlap across genders, stigma surrounding male body image concerns often shapes distinct patterns of symptom expression (Lavender et al., 2017; Rand-Giovannetti et al., 2017).

To date, no studies have examined the psychometric properties of the EDE-QS in Italian samples, despite the availability of an Italian adaptation of the EDE-Q (Calugi et al., 2017). Given the potential impact of cultural factors on symptom expression, establishing the validity and reliability of the Italian EDE-QS is essential for its use in clinical and research contexts. Accordingly, this study aimed to evaluate the psychometric properties of the Italian EDE-QS in a large sample of workers from diverse production sectors undergoing health surveillance at the Catholic University of the Sacred Heart in Rome. Using publicly available data (see Section 2.1), we assessed the measurement model, gender invariance, DIF, and reliability and validity. To advance understanding of the measurement structure and invariance, we employed an Item Response Theory (IRT) framework, applying a two-parameter logistic (2-PL) model (Birnbaum, 1968; Embretson & Reise, 2000) rather than the Rasch model used in the original development.

The 2-PL model offers distinct advantages over the Rasch model for ordinal items such as those in the EDE-QS, primarily by allowing item discrimination to vary. Unlike the Rasch model, which assumes equal discrimination across items, the 2-PL model estimates a discrimination parameter for each item, indicating how effectively it differentiates individuals along the latent trait. This flexibility provides richer insights into item performance and better accommodates the complexity of ordinal data without imposing overly restrictive assumptions (Embretson & Reise, 2000). It is particularly useful when homogeneity in item discrimination cannot be assumed (Crane et al., 2006). Moreover, the 2-PL framework facilitates a more nuanced assessment of measurement invariance, enabling tests of both discrimination and threshold parameters across groups, especially in the case of clinical scales (e.g., Baroni et al., 2022).

Given gender differences in ED prevalence, we examined whether EDE-QS scores differed in their associations with key correlates across men and women. As a first step, we assessed the relationship with age, given prior evidence that adolescents and young adults report higher levels of ED psychopathology, whereas symptom severity tends to decline in older adults (Carrard et al., 2014; Hilbert et al., 2012, 2013). To detect a non-linear association, we also considered age as an ordered factor (Young adulthood [age < 44]; Middle adulthood [45 ≤ age < 65]; Elder [age ≥ 65]). We then examined the association of EDE-QS scores with the Body Mass Index (BMI), both as a continuous variable and as an ordered factor (Underweight [BMI < 18.5], Normal [18.5 ≤ BMI < 24.5], Overweight [24.5 ≤ BMI < 29.5], and Obese [BMI ≥ 29.5]).

The other correlates investigated in this study included indicators of general psychological distress, such as depression and anxiety (e.g., Tan et al., 2023), and of psychological well-being (De Vos et al., 2018; Wade et al., 2012); occupational distress, which can lead to unhealthy coping mechanisms, including disordered eating patterns (Medisauskaite & Kamau, 2019); poor sleep patterns, which can contribute to dysregulated eating behaviors and unhealthy weight management strategies (Kenny et al., 2018); and health literacy and the understanding of nutritional practices, as research has shown that individuals with low health literacy are less likely to engage in healthy eating behaviors, which can increase the risk of disordered eating (Iskender et al., 2024). Moreover, promoting healthy eating literacy in workplace settings can enhance employees’ comprehension of nutritional information, potentially decreasing stress and improving overall health, thereby reducing the incidence of EDs (Agriopoulou & Koutelekos, 2020; Malta et al., 2025). The effects of work-related fatigue, particularly among shift workers, further complicate this landscape, as erratic work hours lead to poor cooking habits and unhealthy dietary choices. This can exacerbate both sleep disturbances and symptoms (Navruz-Varlı & Mortaş, 2024; Olenik & Hećimović, 2024; Sum et al., 2024).

The relationship between workplace violence and the development of eating disorders is an understudied critical area of research, although it has been shown that experiences of violence can significantly impact psychological well-being and behavior. For instance, a study by Brady (2008) indicates that lifetime exposure to family violence is linked to current symptoms of eating disorders in both young men and women, highlighting the role of affective symptoms such as depression and anxiety as mediators in this relationship. These conditions may be exacerbated in work environments where stress and violence are prevalent.

Finally, research indicates that ED symptomatology is associated with increased cigarette smoking as well as problematic alcohol use, suggesting that these behaviors may serve a dual function of weight control and emotional regulation (J. H. Baker et al., 2010, 2018; Munn-Chernoff et al., 2021).

## 2. Materials and Methods

### 2.1. Population

In 2022, in the companies where the Catholic University of Rome (Latium, Italy) carried out health surveillance, employees who were undergoing routine medical examinations for occupational exposure were invited to participate in a health promotion program designed to assess ED symptomatology. Participants with suspected eating disorders were referred to the Italian National Health Service for further diagnostic evaluation and, where appropriate, treatment. However, it should be noted that no diagnostic clinical interview or gold-standard assessment was conducted.

The initiative adopted a cross-sectional census design, inviting all workers to participate voluntarily, without exclusion criteria or active promotion. Participation rates were consistently high in similar programs (Magnavita, 2023), as neither employees nor companies incurred costs; activities fell within the occupational physician’s responsibilities. Participants provided informed consent and authorized the scientific use of their data. Results were anonymized and communicated to employers, company prevention services, and workers’ representatives, accompanied by the occupational physician’s recommendations for workplace preventive measures.

The data collected in the survey were deposited in a publicly available repository (Magnavita, 2025) and secondary data analyses other than the one presented here have already been published (Magnavita et al., 2025). The convenience sample used in this study comprised 1912 participants. There were 1170 (61.2%) women and 742 (38.8%) men, mean age was 45.63 (*SD* = 11.73, range 21–72). These workers were affiliated with various enterprises across sectors, including health (1028, 53.8%), commercial (605, 31.6%), social (172, 9.0%), and industrial (107, 5.6%).

The research received approval from the Ethics Committee of Università Cattolica del Sacro Cuore, Policlinico A. Gemelli, Rome, on 3 March 2022 (ID 4671).

### 2.2. Measures

The Examination Questionnaire, Short Form (EDE-QS, Gideon et al., 2016; the Italian version of the items has been taken from the Italian EDE-Q by Calugi et al., 2017), comprises ten items describing ED-related behaviour and two items concerning negative feelings towards one’s body. The first ten items ask participants to report how often they have engaged in those behaviors over the past seven days on 4-point scale (from “never” = 0 to “6–7 days” = 3), while the last two items require reporting the impact of the negative feelings, still on a 4-point scale (from “not at all” = 0 to “markedly” = 3). The overall score varies from 0 to 36.

Depression and anxiety symptomatology was evaluated using the Italian version (Magnavita, 2007a) of the Goldberg Scale (GADS; Goldberg et al., 1988). Each scale consists of 9 binary questions, with one point awarded for each affirmative response. An individual exhibiting more than five anxiety symptoms or two depressive symptoms has a 50% likelihood of experiencing a clinically significant disruption, with the probability increasing markedly beyond these thresholds.

As a proxy measure for psychological well-being we used Abdel-Khaled’s single-item questionnaire assessing overall life happiness. Participants rated how happy they generally felt at that point in their lives on a scale ranging from 0 to 10. We employed a single-item measure, consistent with most studies in literature (Lukoševičiūtė et al., 2022) and epidemiological research on extensive populations. It has been shown that such measure has a test–retest reliability of 0.86 and adequate criterion and construct validity (Abdel-Khalek, 2006).

The Italian version (Magnavita, 2007b) of Siegrist’s Effort–Reward Imbalance (ERI) questionnaire (Siegrist, 1996) was employed to assess work-related stress. This study employed the abbreviated Italian version of the questionnaire (Magnavita et al., 2012) that comprises 10 items. Three items tap into the effort exerted by workers in their daily job tasks while the remaining seven require the evaluation of the material or immaterial rewards derived from labor. Participants are asked to report their agreement with each item on a 4-point, Likert-type scale (from 1 = “completely disagree” to 4 = “completely agree”). A measure of occupational stress can be obtained as a weighted ratio of the mean score on effort items to the mean score of reward items (Effort/Reward Imbalance Index, ERI). Values exceeding 1 indicate distress.

Sleep quality was assessed using the Italian version (Curcio et al., 2013) of the Pittsburgh Sleep Quality Index (PSQI; Buysse et al., 1989). The PSQI is a self-report instrument comprising Likert-type and open-ended items that evaluate subjective sleep quantity and quality. Item scores range from 0 to 3, with higher scores indicating greater sleep impairment. The questionnaire yields seven component scores (i.e., subjective sleep quality, sleep latency, sleep duration, sleep efficiency, sleep disturbances, use of sleep medication, and daytime dysfunction) which are summed to produce a global PSQI score ranging from 0 to 21.

Health literacy was assessed using the Health Literacy Short Form (HLS-SF12; Finbråten et al., 2018), which consists of 12 selected questions relative to therapies, prevention, and promotion of health from the 47-item European Health Literacy Questionnaire (Sørensen et al., 2013; the Italian version of the items has been taken from the Italian HLS-EU-Q47 by Lorini et al., 2017). Participants were asked to evaluate the ease of performing specific tasks, such as locating information about sickness treatments, using a 4-point Likert-type scale ranging from 1 = “very easy” to 4 = “very difficult”. The overall score may vary from 12 to 48.

The Digital Healthy Diet Literacy survey (DHDLS; Duong et al., 2020) was employed to assess each worker’s proficiency in locating dietary information online. It comprises four items describing the ability to find, process, and implement dietary information. Each item is a 4-point Likert-type scale ranging from 1 = “very easy” to 4 = “very difficult”. The overall score may vary from 4 to 16.

Workers were also queried regarding factors potentially linked to EDs. The study examined carrying out night shifts in the preceding year through a binary yes/no question. The investigation into workplace violence utilized items from Arnetz’s 4-item Violent Incident Form (VIF; Arnetz, 1998) to determine if respondents experienced physical assault, threats, harassment, or persistent and intrusive violence (such as stalking) in the preceding year. Single questions were used to collect data about daily smoking (four categories: “never smoked”, “quit smoking”, “sometimes”, “always”) and alcohol drinking (from 0 = “no unit” to 3 = “2 or more units”).

### 2.3. Data Analysis

Initially, frequency distributions of responses for each EDE-QS item were examined in the total sample and in gender-specific subsamples to verify that all response options were endorsed. The extent of missing data was also evaluated and found to be minimal, with missing responses limited to item 1 (*n* = 2; 0.10%), item 11 (*n* = 1; 0.05%), and item 12 (*n* = 9; 0.47%). Little’s test of missing completely at random was significant (χ^2^(33) = 130.39, *p* < 0.001). However, in large samples like the one in this work, this test will almost inevitably be significant, even when the departure from MCAR is trivially small and attributable to a missing at random (MAR) mechanism(s) of no practical consequence for inference (see, e.g., Sondhi et al., 2023; Thakur et al., 2023). Given the negligible proportion of missing values and the absence of compelling substantive reasons to believe that missingness depends on unobserved values, we concluded that the missing data could be considered as MAR. Under MAR, multiple imputation and other modern missing data methods yield valid inferences (see, e.g., Escobar et al., 2020; Steinsland et al., 2014). Hence, missing data were imputed using the *imputeMissing* function from the *mirt* package (version 1.45.1; Chalmers, 2012) in R (version 4.4.2; R Core Team, 2024).

The measurement model for the EDE-QS items was specified using the graded response model (GRM; Samejima, 1969, 1997). The GRM is a two-parameter logistic model suitable for items with two or more ordered response categories, such as those of the EDE-QS. It estimates an item-specific discrimination (*slope*) parameter and a set of *k* − 1 threshold parameters corresponding to the boundaries between adjacent response categories.

As with all item response theory (IRT) models, accurate estimation of graded response model (GRM) parameters depends on the extent to which the assumption of unidimensionality is satisfied (i.e., whether a single latent trait accounts for the observed item responses). As noted by Reise et al. (2015), psychological constructs are often defined by multiple, conceptually diverse indicators, and content-valid measurement therefore entails heterogeneous item pools. Consequently, strict unidimensionality is rarely achieved in practice; however, measures may still be considered sufficiently unidimensional for IRT modeling purposes. This issue has been discussed in detail by Reise et al. (2015) and Rodriguez et al. (2016a, 2016b).

The dimensionality and factor structure of the EDE-QS item pool were examined in the total sample and in gender-specific subsamples using multiple complementary methods. To determine whether a unidimensional or multidimensional measurement model was more appropriate, we first estimated the optimal number of factors using the scree test (Cattell, 1966), parallel analysis (PA; Buja & Eyuboglu, 1992; Horn, 1965; Longman et al., 1989), and the minimum average partial (MAP) correlation statistic (Velicer, 1976). The rationale for employing these methods is detailed in Supplementary Materials S1. These analyses were conducted using the *fa.parallel* and *vss* functions from the *psych* package (version 2.4.6.26; Revelle, 2024) in R. When evidence supported a multidimensional solution, exploratory multidimensional item response theory (E-MIRT) models were estimated to identify multiple latent traits underlying item responses without imposing a priori constraints on the factor structure. *E-MIRT* is well suited for binary or ordinal items and employs full-information maximum likelihood estimation, yielding likelihood-based fit indices (Reckase, 2009). This analysis was performed using the *mirt* function in the *mirt* package (version 1.45.1; Chalmers, 2012) in *R*.

Second, graded response models (GRMs) were fitted to the full sample and to gender-specific subsamples using the *mirt* function from the *mirt* package (version 1.45.1; Chalmers, 2012) in *R*. Unidimensionality was preliminarily evaluated by examining item loadings on the single latent factor, with loadings exceeding 0.30 indicating that the factor accounted for at least 10% of item variance. In addition, three recommended unidimensionality indices (Rodriguez et al., 2016a, 2016b) were computed using dedicated functions from the *BifactorIndicesCalculator* package (version 0.2.2; Dueber, 2021). Composite reliability was assessed using McDonald’s *omega* (McDonald, 1999), an index appropriate for summed total scores with equally weighted items and shown to provide a more accurate estimate of internal consistency than Cronbach’s alpha, as it does not assume tau-equivalence and accounts for variance attributable to all latent factors (Bentler, 2007; McNeish, 2018). Omega coefficients greater than 0.80 were interpreted as a necessary, though not sufficient, condition for unidimensionality (Rodriguez et al., 2016a, 2016b). Factor score determinacy (FSD) represents the multiple correlation between observed variables and their corresponding latent factor (Grice, 2001), indexing the proportion of factor variance captured by the estimated factor scores. Values exceeding 0.90 indicate adequate unidimensionality and support the validity of factor score use (Gorsuch, 1983). Finally, construct replicability was evaluated using the *H* index (Hancock & Mueller, 2001), which reflects the extent to which a latent variable is well defined by its indicators and is expected to replicate across studies. Values of 0.70 or higher were considered adequate. Confidence intervals for all indices were estimated via bootstrapping.

Model goodness of fit was evaluated using the *C*_2_ statistic (Cai & Monroe, 2014), an omnibus limited-information fit index with an approximate χ^2^ distribution. Like the traditional χ^2^ statistic, *C*_2_ is sensitive to sample size and may therefore reject well-fitting models in large samples. The *M*_2_ function in the *mirt* package additionally provided SEM-based fit indices derived from the *C*_2_ statistic, including the comparative fit index (CFI_C2_), Tucker–Lewis index (TLI_C2_) and the root mean square error of approximation (RMSEA_C2_) with 95% confidence intervals. Because no formal cutoff guidelines exist for *C*_2_-based indices, commonly used structural equation modeling (SEM) criteria were applied for heuristic evaluation of single-model fit (RMSEA ≤ 0.06/0.08 and CFI/TLI ≥ 0.95/0.90 for excellent/acceptable fit, respectively; Marsh et al., 2004), with due caution in their interpretation. Item-level fit was assessed using the *S-X*^2^ statistic (Orlando & Thissen, 2003), computed via the *itemfit* function in the *mirt* package (version 1.45.1; Chalmers, 2012), which has shown adequate performance with the GRM (Kang & Chen, 2011).

As a check for parsimony, we also tested a “reduced” model in which discrimination parameters were constrained to be equal for all items (hence a 1-PL model), and compared its fit with the 2-PL GRM. The relative fit of the GRM and reduced GRMs was evaluated using a likelihood-ratio test based on the deviance statistic, defined as the difference in −2 log-likelihood (−2LL) between models. This statistic follows a χ^2^ distribution with degrees of freedom equal to the difference in the number of estimated parameters between the full GRM (with item-specific discrimination parameters) and the reduced model (with a single discrimination parameter). Model comparisons were further informed by information criteria, including the Akaike Information Criterion (AIC; Akaike, 1974), the Bayesian Information Criterion (BIC; Schwarz, 1978), and the sample-size adjusted BIC (SABIC; Sclove, 1987). Although these indices do not provide absolute measures of model fit, lower values indicate superior relative fit. Therefore, they were used to compare the GRM and its reduced version and the measurement invariance models described below. Caution should also be exercised in considering these indices, since, with sufficiently large sample sizes, they tend to support more complex alternatives (see Marsh et al., 2005).

The same goodness-of-fit criteria were applied to evaluate measurement invariance of the EDE-QS across gender. Model comparisons were conducted using chi-square difference tests; however, given their sensitivity to sample size, changes in approximate fit indices were also considered. Consistent with recommendations in the SEM literature, invariance was supported when changes in the CFI were smaller than 0.01 and changes in the RMSEA were smaller than 0.015 (Chen, 2007; Cheung & Rensvold, 2002).

Measurement invariance across gender was evaluated using a sequence of nested models. First, a configural invariance model (Model 1) was specified, in which all discrimination (slope) and threshold parameters were freely estimated across groups. Next, a metric invariance model (Model 2) was estimated by constraining discrimination parameters to equality across groups. Finally, a scalar invariance model (Model 3) was tested, imposing equality constraints on both discrimination parameters and thresholds. This model permits valid comparisons of latent means and the evaluation of differential item functioning (DIF), defined as group differences in response probabilities among individuals with equivalent levels of the latent trait. Evidence of DIF across all items would invalidate latent mean comparisons, whereas partial invariance, which is expected when thresholds are invariant for only a subset of items, is sufficient for meaningful inference. Following Byrne et al. (1989), invariance of at least two item thresholds per latent trait was considered adequate for latent mean estimation.

To identify items exhibiting substantial DIF, we applied the procedure proposed by Meade and Wright (2012). Starting from the full scalar invariance model, likelihood ratio tests (LRTs; Thissen et al., 1988, 1993) were used to sequentially free item parameters while treating the remaining items as anchors. Five items with nonsignificant LRTs and the largest discrimination parameters were selected as anchor items according to the A5 method, and a partial scalar invariance model (Model 3p) was subsequently estimated. Because LRTs may detect trivial DIF in large samples, item-level DIF was additionally evaluated using the Expected Score Standardized Difference (ESSD), interpreted according to Cohen’s (1988) benchmarks for effect size (i.e., |*d*|< 0.20 negligible effect; 0.20 ≤ |*d*| < 0.50: small effect; 0.50 ≤ |*d*| < 0.80: moderate effect; |*d*| ≥ 0.80 large effect).

Finally, we tested whether the correlation of the EDE-QS score with the other variables of interest differed between women and men using a test for the comparison of independent correlation coefficients (Fisher, 1925), as implemented in the *cocor.indep.groups* function in the *cocor* package (version 1.1-4; Diedenhofen & Musch, 2015) in *R*. Correlations between the EDE-QS score and metric variables were computed as Pearson’s correlation coefficient using the *cor.test* function of the *stats* basic package in *R*. Correlations between the EDE-QS score and categorical variables were computed as the square root of the *eta-square* effect size of analysis of variance (ANOVA) using the *eta*_*squared* function in the *effectsize* package (version 1.0.1; Ben-Shachar et al., 2020) in *R*. An ANOVA was performed, and, if it was significant, model-based least squares pairwise comparisons of marginal means using the so-called “usual-t” tests (Toothaker, 1993, as implemented in the *pairs* function in the *emmeans* package, version 1.10.5, Lenth, 2024) were carried out, with false discovery rate correction of the *p*-values to control for the inflation of type I error due to multiple comparisons. Correlations between the EDE-QS score and ordinal variables were computed as polyserial correlations using the *polyserial* function in the *polycor* package (version 0.8-1; Fox, 2022) in *R*.

## 3. Results

### 3.1. Frequency Distribution of EDE-QS

**Figure 1:**
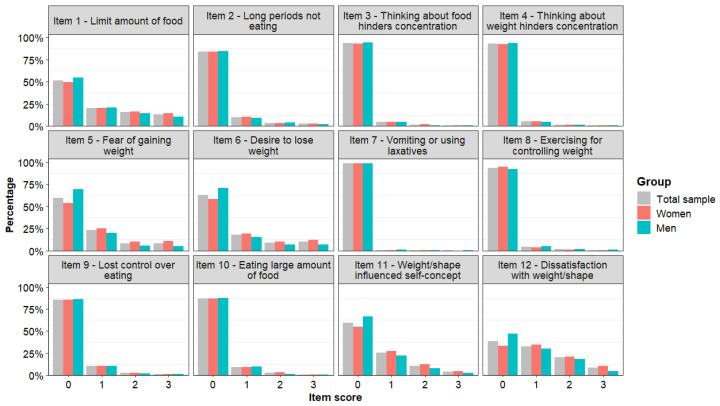
Score distribution of EDE-QS items.

We initially inspected the frequency distribution of the four possible answers to the EDE-QS items (Figure 1).

All items showed a substantial positive skewness, particularly item 7. The details of frequencies and percentages are reported in Supplementary Material S2.

### 3.2. EDE-QS Dimensionality and Measurement Model

**Figure 2:**
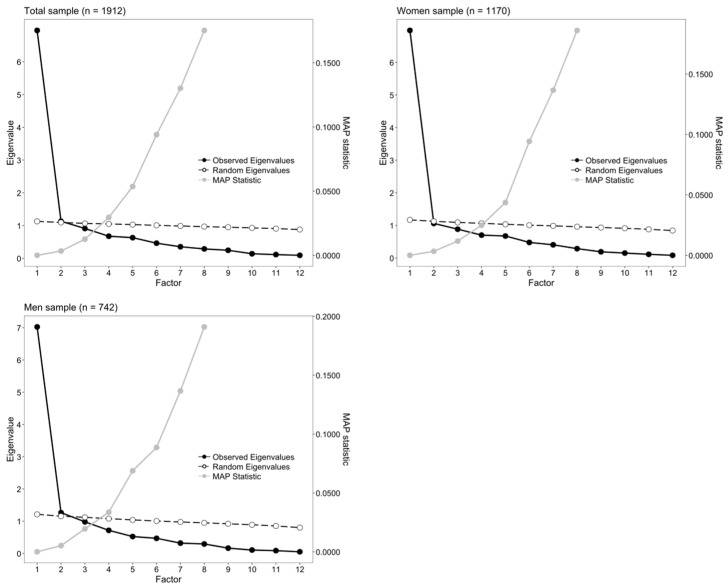
Scree-plots, results from the parallel analysis and the Minimum Average Partial (MAP) correlation statistic for the EDE-QS in total sample and in the subsamples defined by gender.

We then investigated the dimensionality of the EDE-QS in the total sample and in the subsamples defined by gender. The results of the scree test, the PA, and the MAP are shown in Figure 2. In all cases, we found evidence of a strong first factor, which explained at least 58% of variance. The descending line of the eigenvalues started to flatten out from the second factor, and the MAP values reached their minimum with one factor. However, there was some evidence of multidimensionality, as the parallel analysis suggested that the optimal number of factors could be two in the total and in the men sample.

We thus tested a two-factor model in all samples using E-MIRT. According to the criteria described in Section 2.3, the two-factor models had a better fit than the unidimensional models (Table 1), but did not show evidence of ‘approximate simple structure’ (Sass & Schmitt, 2010), that is, a solution with all items substantially loading (>|0.30|) on only one factor, with near-zero cross-loadings, and with factors defined by at least three indicators. Instead, we found that in all solutions there was at least one item with more than one substantial loading, and the factor correlations exceeded 0.70, suggesting redundancy (see Supplementary Material S3 for details). Taking into account that the loadings on the first factor were all larger than 0.60 (see Supplementary Material S3), that the second factor explained at best 10.5% of variance, and that the first-to-second eigenvalue ratios were well over 4 (Slocum-Gori & Zumbo, 2011), we concluded that these results supported approximate unidimensionality sufficient for IRT modeling.

The unidimensional GRM also proved to have a better fit than the reduced model (1-PL), suggesting that the assumption of tau-equivalence was not adequately supported (loadings ranged from 0.60 to 0.90, see Supplementary Material S3).

Parameter estimates for the GRM are reported in Supplementary Material S4. According to F. B. Baker (2001), discrimination parameters exceeding 0.65 indicate adequate differentiation between low and high levels of the latent trait; this criterion was surpassed by all items across samples. Although several items yielded statistically significant S-X^2^ statistics following Benjamini–Hochberg false discovery rate correction (Benjamini & Hochberg, 2000), likely reflecting the large sample size, associated RMSEA values were consistently below 0.05. These results indicate satisfactory model fit across samples (see Supplementary Material S5).

The indices of unidimensionality were adequate in all samples. In the total sample *omega* was 0.949 [0.942; 0.951], FSD was 0.981 [0.979; 0.981], and *H* was 0.962 [0.959; 0.962]. In the women sample these values were 0.950 [0.940; 0.957], 0.979 [0.976; 0.983], and 0.959 [0.953; 0.966], respectively. In the men sample these values were 0.951 [0.940; 0.959], 0.983 [0.980; 0.987], and 0.967 [0.961; 0.974], respectively.

Taken together, these results can be considered as support for approximate unidimensionality sufficient for IRT modeling in the total sample and in the subsamples defined by gender.

### 3.3. EDE-QS Invariance Across Gender

When we tested the measurement invariance across gender, we found support for configural (Model 1) and metric (Model 2) invariance but not for scalar invariance (Table 1). Model 2 had a CFI that was more than 0.01 smaller than the CFI of Model 1, but the chi-square difference was not statistically significant. Considering the sensitivity of this test to sample size, we interpreted this result as support for metric invariance (i.e., invariance of slopes). Moreover, when we tested the invariance of each slope, none of them were statistically significant (see Supplementary Material S6).

**Figure 3:**
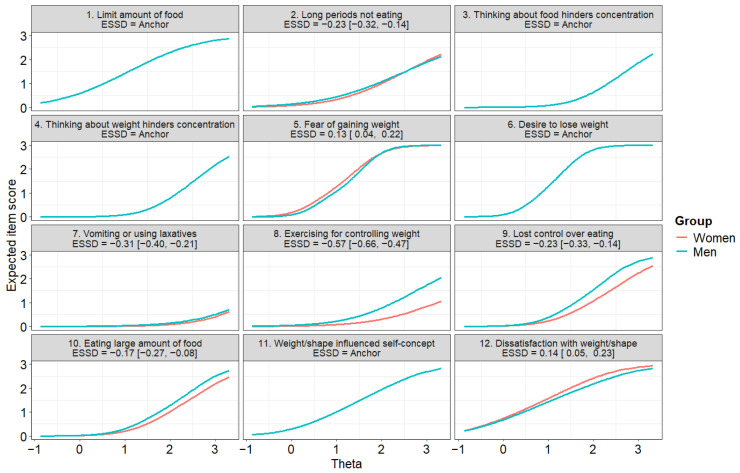
Expected Score Standardized Difference (ESSD) and Differential Item Functioning of the EDE-QS across gender. Negative values indicate higher expected scores in men.

The drop in CFI from the metric to the scalar invariance model (Model 3) was larger than 0.01 and the chi-square test was statistically significant (Table 1). Therefore, we ran a DIF analysis and identified the five no-DIF items with the largest slopes, which were used as anchors (items 1 [‘Limit amount of food’], 3 [‘Thinking about food hinders concentration’], 4 [‘Thinking about weight hinders concentration’], 6 [‘Desire to lose weight’], and 11 [‘Weight/shape influenced self-concept’]). We then tested a partial invariance model (Model 3p), whose fit was adequate and not substantially different from that of Model 2 (Table 1). This model also allowed us to test the latent mean difference, which was statistically significant, with higher scores in women but a small effect size (estimate = 0.34, standard error = 0.06, *z* = 5.91, *p* < 0.001; *d* = 0.35 [0.25, 0.44]). ESSDs for the EDE-QS items are reported in Figure 3.

We observed a moderate DIF for item 8 (‘Exercising for controlling weight’) and small DIFs for items 2 (‘Long periods not eating’), 7 (‘Vomiting or using laxatives’), and 9 (‘Lost control over eating’). Notably, in all these items, men tended to show higher expected scores than women with the same trait level. In all other items, the DIF was negligible.

### 3.4. Correlates of EDE-QS Across Gender

Table 2 reports the descriptive statistics by gender for the other variables considered in this study, the correlations of EDE-QS with these, and the results of their comparisons across gender. No comparison of correlations was significant after adjustment of the *p*-values for multiple comparisons, and effect sizes were all negligible (*r* < |0.10|), suggesting that the pattern of association of EDE-QS with the other variables did not differ between women and men.

Table 2 also shows that there were significant and moderate (0.30 ≤ |*r*| < 0.50) positive correlations of the EDE-QS total score with BMI, depression, anxiety, and PSQI. Other correlations, such as those with happiness, reward, and ERI, were statistically significant despite the small effect size (0.10 ≤ |*r*| < 0.30), due to the large sample size.

## 4. Discussion

This study evaluated the psychometric properties of the Italian version of the Eating Disorder Examination Questionnaire—Short Form (EDE-QS; Gideon et al., 2016) in a large sample of workers undergoing health surveillance. Using an Item Response Theory framework, we examined dimensionality, reliability and measurement invariance across gender, and we tested associations with relevant correlates. Results supported an approximate unidimensional structure sufficient for IRT modeling, consistent with previous validations (Duffy et al., 2021; Gideon et al., 2016; He et al., 2021; Mousavi Asl et al., 2021; Prnjak et al., 2020). Internal consistency was excellent (ω ≈ 0.95; McDonald, 1999), and indices of factor score determinacy and construct replicability exceeded recommended thresholds (Hancock & Mueller, 2001). Measurement invariance analyses indicated configural and metric invariance but not full scalar invariance, due to differential item functioning (DIF) in a subset of items. Latent mean differences were small, with women scoring slightly higher than men, in line with prior evidence on gender differences in ED symptomatology (Lavender et al., 2017; Schaefer et al., 2018, 2019).

The findings indicate that the Italian EDE-QS is a psychometrically robust instrument for assessing ED symptoms in occupational settings. Strong unidimensionality indices and high discrimination parameters suggest that the scale mainly captures a single latent construct, consistent with the Rasch-based unidimensional structure reported in cross-cultural adaptations (Dahlgren et al., 2025; He et al., 2021). Reliability met conventional standards (Rodriguez et al., 2016b, 2016a), supporting the use of sum scores for research and for the dimensional assessment of ED symptom severity.

The lack of scalar invariance seems to reflect gender-specific symptom expression documented in previous studies (Lavender et al., 2017; Rand-Giovannetti et al., 2017). The detection of DIF in items assessing behaviors such as exercising to control weight, prolonged fasting, vomiting or laxative use, and loss of control on eating provides important insights into this issue. Notably, men exhibited higher expected scores than women at equivalent levels of the latent construct, suggesting that these behaviors may hold greater salience in male presentations of ED symptomatology. This pattern is consistent with prior research indicating that men often engage in compensatory behaviors, particularly excessive exercise, as part of weight-control strategies, likely reflecting sociocultural ideals that emphasize muscularity and leanness rather than thinness (Lavender et al., 2017; Schaefer et al., 2018, 2019). Such behaviors may be perceived as socially acceptable or even desirable in male populations, potentially contributing to under-recognition of disordered eating in men (Kinasz et al., 2016; Rand-Giovannetti et al., 2017). Future research might consider developing additional items that better capture muscular dysmorphia or compensatory exercise behaviors in men.

The presence of DIF in items related to purging and fasting behaviors, although traditionally associated with female profiles, suggests that these strategies are not absent among men but may manifest differently or in response to distinct body image concerns. Previous studies have highlighted that while core symptoms overlap across genders, men often report patterns linked to muscularity-oriented goals rather than weight loss *per se* (Lavender et al., 2017; Schaefer et al., 2018, 2019). This divergence underscores the complexity of assessing ED symptoms in men and the limitations of instruments developed and phrased primarily for female populations (Thielemann et al., 2019). Furthermore, stigma surrounding male body image concerns and reluctance to endorse conventional diagnostic labels may lead men to emphasize behaviors such as exercise while minimizing other symptoms, complicating clinical detection (Rand-Giovannetti et al., 2017).

From a measurement perspective, these findings highlight the importance of considering gender-related item bias when interpreting EDE-QS scores. Although the overall impact of DIF on total scores was modest, failure to account for such differences could obscure meaningful variations in symptom profiles and perpetuate gender disparities in diagnosis and treatment, supporting the critical demand for tailored assessments that accurately reflect diverse gender experiences in EDs (Brasil et al., 2023). Future research should explore whether item-level adjustments or gender-specific norms can enhance score interpretability and improve the instrument’s potential utility in applied and clinical contexts. Clinically, practitioners should be aware that elevated scores on exercise-related items may signal significant pathology in men, even in the absence of high endorsement of traditional ED behaviors.

Nevertheless, metric invariance supports the meaningful comparison of associations with external variables across genders. In this study, we observed no significant gender differences in these associations, indicating that, despite minor gender-related item functioning differences, the overall relationship between ED symptomatology and psychological or occupational factors remains largely invariant. These findings suggest that mechanisms linking ED symptoms to correlates such as mood disturbances (Tan et al., 2023), sleep impairment (Kenny et al., 2018), and occupational stress (Medisauskaite & Kamau, 2019) operate similarly in men and women. Consistent with prior research, our results imply that while gender may influence prevalence and initial symptom expression, its impact on broader psychological health associations diminishes substantially (Coelho et al., 2021; Joy et al., 2022; Tomba et al., 2014). Practically, interventions addressing psychological distress, sleep problems, and health literacy appear equally relevant for both genders in workplace contexts.

Some limitations need to be acknowledged. First, the cross-sectional design precluded the assessment of temporal stability and predictive validity (Hilbert et al., 2012). Second, although the sample enrolled for this study was large and heterogeneous and included workers from multiple sectors, it was drawn from a single cultural context, limiting cross-cultural generalizability (Costello et al., 2023; Lichtenstein et al., 2021). Half of the sample was composed of healthcare professionals, raising the possibility of bias given their distinctive stress profiles and health-related expertise. To address this concern, we fitted a general linear model examining the association between EDE-QS scores and professional sector while controlling for age, gender, and their interactions with sector. The analysis revealed no significant main effect of professional sector and no significant interaction terms, indicating that professional sector did not meaningfully influence EDE-QS scores. Fourth, participants were not formally evaluated for an ED diagnosis, and this did not allow us to test the criterion validity and screening efficacy of the questionnaire and to compute diagnostic cut-off scores. While the current findings provided evidence of robust psychometric properties (structural validity, reliability, and measurement invariance) in a non-clinical population, the lack of clinical data limits conclusions regarding the questionnaire’s ability to accurately differentiate individuals with ED from those without. For descriptive purposes, we report EDE-QS total scores corresponding to 2 and 3 standard deviations above the mean (Women: 16.01 and 21.40, respectively; Men: 13.62 and 18.45, respectively), the 95th, 97.5th, and 99th percentile (Women: 16.00, 19.00, and 23.00, respectively; Men: 14.00, 17.00, and 21.59, respectively). These figures should not be interpreted as diagnostic or screening cut-offs, but rather as distribution-based reference points within this non-clinical sample. Future research should incorporate clinically diagnosed participants to enable rigorous validation against gold-standard diagnostic criteria and to determine optimal thresholds for screening purposes. Such work is essential to ensure the tool’s applicability in both research and clinical settings.

Occupational medicine practice may benefit from the availability of a brief and psychometrically sound instrument for assessing ED symptomatology. In workplace health surveillance, such a tool could facilitate the identification of individuals reporting elevated symptom levels, who may warrant further clinical evaluation through appropriate diagnostic pathways. This immediate intervention has no cost to the worker or the company and allows the occupational physician to monitor the patient’s progress in the context of the therapeutic intervention. Furthermore, the collective analysis of data obtained from health surveillance can motivate companies to invest in the promotion of healthy eating to prevent the impact that EDs can have on productivity.

## 5. Conclusions

The Italian EDE-QS appears suitable for the rapid assessment of ED symptomatology in workplace health surveillance contexts (Murray et al., 2025). Its brevity and strong psychometric properties make it an efficient tool for occupational physicians and mental health professionals (Gideon et al., 2016). When elevated symptom levels are detected, referral for specialist assessment may facilitate the identification and treatment of clinically confirmed EDs, thus reducing the underdiagnosis of these disorders, at least two-thirds of which are untreated (Keski-Rahkonen & Mustelin, 2016). The findings also contribute to the literature on gender differences in ED symptomatology (Lavender et al., 2017; Schaefer et al., 2018, 2019), highlighting the need for nuanced interpretation of specific items. Organizations may consider the EDE-QS as a structured tool for monitoring ED symptom levels within employee populations, which could inform preventive initiatives when interpreted cautiously and in conjunction with appropriate clinical expertise, thereby promoting well-being and productivity (Ubhi et al., 2025). Further longitudinal research conducted in collaboration between companies and the National Health Service will be able to define the clinical evolution of suspected cases. However, practitioners should be aware of potential gender-related response patterns when interpreting individual scores.

## Figures and Tables

**Table 1:** Fit indices of the single sample and invariance Graded Response Models (GRM).

Model	*M*_2_	df	*p*	CFI	TLI	RSMEA [95% CI}	AIC	BIC	SABIC	LL	Δχ^2^	Δdf	Δ*p*
Total sample (n = 1912)													
GRM (single-factor)	2780.56	30	<0.001	0.968	0.955	0.066 [0.059, 0.073]	258,420.75	261,090.43	259,560.94	−128,730.38			
Two-factor	750.85	19	<0.001	0.993	0.984	0.040 [0.030, 0.049]	255,010.58	258,290.38	256,410.94	−126,910.79	3630.17 °	11	<0.001
Reduced	5780.89	41	<0.001	0.930	0.928	0.083 [0.077, 0.089]	261,800.88	263,860.45	262,680.90	−130,530.44	3600.13 °	11	<0.001
Women (n = 1170)													
GRM (single-factor)	2130.61	30	<0.001	0.959	0.942	0.072 [0.063, 0.082]	165,470.77	167,900.88	166,380.41	−82,250.89			
Two-factor	510.68	19	<0.001	0.993	0.984	0.038 [0.026, 0.051]	163,430.49	166,420.31	164,540.91	−81,120.75	2260.28 °	11	<0.001
Reduced	4060.24	41	<0.001	0.918	0.916	0.087 [0.080, 0.095]	166,990.97	168,870.37	167,690.85	−83,120.99	1740.20 °	11	<0.001
Men (n = 742)													
GRM (single-factor)	1100.57	30	<0.001	0.976	0.966	0.060 [0.048, 0.072]	92,210.70	94,420.95	92,900.53	−45,620.85			
Two-factor	480.65	19	<0.001	0.991	0.980	0.046 [0.030, 0.062]	91,020.80	93,740.75	91,870.41	−44,920.40	1400.90 °	11	<0.001
Reduced	2530.65	41	<0.001	0.937	0.935	0.084 [0.074, 0.094]	93,880.63	95,590.17	94,410.69	−46,570.31	1880.93 °	11	<0.001
Invariance models													
Model 1—configural	3240.13	60	<0.001	0.975	0.965	0.048 [0.043, 0.053]	257,690.47	263,020.83	259,970.84	−127,880.73			
Model 2—Weak/metric	4400.36	72	<0.001	0.965	0.959	0.052 [0.047, 0.056]	257,600.16	262,260.85	259,590.98	−127,960.08	140.69	12	0.259
Model 3—Strong/scalar	5980.04	106	<0.001	0.953	0.963	0.049 [0.045, 0.053]	258,020.93	260,800.72	259,210.87	−128,510.46	1100.77	34	<0.001
Model 3p—Partial strong/scalar	4740.97	78	<0.001	0.962	0.959	0.052 [0.047, 0.056]	257,520.96	261,860.32	259,380.52	−127,980.48	40.81 ^	6	0.569

*Note*: *M*_2_: model statistic; df: degrees of freedom; *p*: *p*-value; CFI: Comparative Fit Index; TLI: Tucker–Lewis Index; RMSEA [95% CI]: Root Mean Square Error of Approximation and its 95% confidence interval; AIC: Akaike Information Index; BIC: Bayesian Information Index; SABIC: Sample-size Adjusted BIC; LL: log-likelihood; Δχ^2^ = −2 × (LL_restricted_ − LL_null_); Δdf: df_restricted_ − df_null_; Δ*p*: *p*-value for Δχ^2^; °: tested against GRM; ^: tested against Model 2. AIC and BIC values are reported for relative model comparison; their absolute magnitude increases with sample size because they are based on the −2*log-likelihood. With large sample sizes *n*s, small improvements in fit can produce sizeable changes in information criteria. AIC applies a constant complexity penalty (2*k*) and may therefore favor more complex models more readily, whereas BIC applies a sample-size–dependent penalty (*k* log *n*) and becomes increasingly conservative as *n* increases. Accordingly, one should interpret differences in AIC/BIC across models (not the absolute values) when evaluating model parsimony.

**Table 2:** Descriptive statistics and bivariate correlations EDE-QS scores with the other variables in women and men. Coefficients are Pearson correlations unless otherwise indicated. More details are reported in Supplementary Material S7.

Variable	Women		Men		Z	*p*	adj-*p*	Effect Size *r*
	Descriptives	Correlation	Descriptives	Correlation				
Age	44.61 ± 11.75	0.104 ***	47.24 ± 11.54	0.018	−1.822	0.069	0.393	−0.086 [−0.130, −0.041]
Age categorical ^a^		0.122 ***		0.060	−1.324	0.185	0.393	−0.062 [−0.107, −0.017]
age < 44	574 (49.1%)		317 (42.7%)					
45 ≤ age < 65	565 (48.3%)		378 (50.9%)					
age ≥ 65	31 (2.6%)		47 (6.3%)					
BMI	23.97 ± 4.58	0.387 ***	25.92 ± 4.19	0.328 ***	−1.453	0.146	0.393	−0.068 [−0.113, −0.024]
BMI categorical ^a^		0.363 ***		0.285 ***	−1.838	0.066	0.393	−0.086 [−0.131, −0.042]
BMI < 18.5	52 (4.4%)		6 (0.8%)					
18.5 ≤ BMI < 24.5	531 (45.4%)		258 (34.8%)					
24.5 ≤ BMI < 29.5	227 (19.4%)		278 (37.5%)					
BMI ≥ 29.5	109 (9.3%)		95 (12.8%)					
Depression	1.98 ± 2.26	0.377 ***	1.33 ± 1.96	0.351 ***	−0.634	0.526	0.745	−0.030 [−0.075, 0.015]
Anxiety	3.11 ± 2.72	0.380 ***	2.08 ± 2.40	0.318 ***	−1.496	0.135	0.393	−0.070 [−0.115, −0.026]
Happiness	7.06 ± 1.76	−0.290 ***	7.22 ± 1.84	−0.259 ***	0.732	0.464	0.717	0.034 [−0.010, 0.079]
Effort	7.03 ± 2.30	0.155 ***	7.15 ± 2.41	0.161 ***	0.135	0.893	0.955	0.006 [−0.039, 0.051]
Reward	19.88 ± 3.69	−0.203 ***	19.75 ± 3.88	−0.189 ***	0.307	0.759	0.922	0.014 [−0.030, 0.059]
ERI	0.88 ± 0.40	0.215 ***	0.91 ± 0.45	0.209 ***	−0.128	0.899	0.955	−0.006 [−0.051, 0.039]
PSQI	6.13 ± 3.48	0.349 ***	5.49 ± 3.07	0.316 ***	−0.789	0.430	0.717	−0.037 [−0.082, 0.008]
HLS-SF12	37.26 ± 5.20	−0.155 ***	37.54 ± 6.18	−0.211 ***	−1.253	0.210	0.397	−0.059 [−0.103, −0.014]
DHDLS	11.22 ± 2.77	−0.038	11.34 ± 3.13	−0.120 **	−1.770	0.077	0.393	−0.083 [−0.128, −0.039]
Night work		−0.008		−0.076 *	−1.463	0.144	0.393	−0.069 [−0.113, −0.024]
No	557 (75.1%)		856 (73.2%)					
Yes	185 (24.9%)		314 (26.8%)					
Violence	0.25 ± 0.64	0.123 ***	0.21 ± 0.64	0.143 ***	0.441	0.660	0.863	0.021 [−0.024, 0.065]
Smoking ^a^		0.018		0.019	0.034	0.973	0.973	0.002 [−0.043, 0.046]
Never	560 (47.86%)		321 (43.3%)					
Quit	201 (17.18%)		175 (23.6%)					
Sometimes	135 (11.54%)		86 (11.6%)					
Always	273 (23.33%)		159 (21.4%)					
Drinking ^b^		0.048		−0.016	−1.367	0.172	0.393	−0.064 [−0.109, −0.020]
No unit	729 (62.3%)		309 (41.6%)					
One unit	396 (33.8%)		371 (50.0%)					
Two units	35 (3.0%)		53 (7.1%)					
Two or more units	10 (0.9%)		7 (0.9%)					

*Note*: Descriptive statistics are mean ± standard deviation or frequency with column percentage; Z: Z-statistic; *p*: *p*-value; adj-*p*: False Discovery Rate-adjusted *p*-value; *r*: effect size for the difference between independent correlation coefficients; BMI: Body Mass Index; ERI: Effort/Reward Imbalance Index; PSQI: Pittsburgh Sleep Quality Index; HLS-SF12: Health Literacy Short Form; DHDLS: Digital Healthy Diet Literacy Survey; ^a^: square root of ANOVA’s eta-square effect size; ^b^: polyserial correlation. * *p* < 0.05; ** *p* < 0.01; *** *p* > 0.001.

## Data Availability

Data are deposited on Zenodo https://doi.org/10.5281/zenodo.15685523 (Uploaded on 17 June 2025).

## Supplementary Materials

The following supporting information can be downloaded at: https://www.mdpi.com/article/10.3390/ejihpe16030037/s1, Supplementary Material S1: Rationale of the dimensionality tests; Supplementary Material S2: Frequencies and percentages of score endorsement. Supplementary Material S3: Factor loading matrices. Supplementary Material S4: Parameter estimates; Supplementary Material S5: Orlando and Thissen’s (2003) S-X^2^ item fit indices, Supplementary Material S6: Slopes modification indices for the metric invariance model. Supplementary Material S7: Details of the correlational analyses.

## Abbreviations

The following abbreviations are used in this manuscript:

1-PL	One-parameter logistic model
−2LL	−2 log likelihood
2-PL	Two-parameter logistic model
AIC	Akaike Information Criterion
AN	Anorexia nervosa
ANOVA	Analysis of variance
BED	Binge eating disorder
BIC	Bayesian Information Criterion
BMI	Body mass index
BN	Bulimia nervosa
CFI	Comparative fit index
CI	Confidence interval
df	Degrees of freedom
DHDLS	Digital Healthy Diet Literacy survey
DIF	Differential item functioning
DSM-5-TR	Diagnostic and Statistical Manual of Mental Disorders, 5th edition, Text Revision
ED	Eating disorder
EDE	Eating Disorder Examination
EDE-Q	Eating Disorder Examination-Questionnaire
EDE-QS	Eating Disorder Examination-Questionnaire—Short Form
ERI	Effort–Reward Imbalance questionnaire
ESSD	Expected Score Standardized Difference
FSD	Factor score determinacy
GADS	Goldberg Depression/Anxiety Scale
GOF	Goodness of fit
GRM	Graded response model
HLS-EU-Q47	European Health Literacy Questionnaire
HLS-SF12	Health Literacy Scale-Short Form
ICD-11	International Classification of Diseases, 11th edition
IRT	Item Response Theory
LRT	Likelihood ratio test
MAP	Velicer’s minimum average partial correlation statistic
PA	Parallel analysis
PSQI	Pittsburgh Sleep Quality Index
RMSEA	Root mean square error of approximation
SABIC	sample size-adjusted Bayesian Information Criterion
SEM	Structural equation modeling
TLI	Tucker–Lewis index
VIF	Violent Incident Form
WHO	World Health Organization
