# Supplementary material for: Factor Structure and Measurement Invariance Across Gender of the Eating Disorder Examination Questionnaire—Short Form in Italian Workers
Source: Eur J Investig Health Psychol Educ. 2026 Mar 5;16(3):37. doi: 10.3390/ejihpe16030037 (PMC13025737; doi:10.3390/ejihpe16030037)
Supplement: Supplementary file 1 [file ejihpe-16-00037-s001.zip › ejihpe-4109835-supplementary.pdf]

European Journal of
Investigation in Health, Psychology and Education

Supplementary Materials for the article:
Factor structure and measurement invariance across gender of the Eating Disorder Examination Questionnaire-Short Form in Italian workers

Nicola Magnavita and Carlo Chiorri

S1. Rationale of the dimensionality tests

The scree test (Cattell, 1966) involves a visual examination of a dot graph where the eigenvalues derived from factor analysis are placed against their corresponding factors in the order of extraction, connected by a straight line. The slope of the line linking two consecutive dots diminishes progressively as the number of factors increases. The optimal number of factors is determined at the juncture where the curve starts to level out. As a result, unidimensionality would be corroborated by a scree plot that levels out after the second factor. The scree test has frequently faced criticism for its subjective determination of the cutoff between significant and trivial factors, prompting the suggestion of other analytical and objective ways to assess the dimensionality of an item pool.

Parallel analysis (Horn, 1965) involves deriving eigenvalues from randomly generated correlation matrices (usually 1,000; Buja & Eyuboglu, 1992) that match the original dataset in terms of the number of variables and cases. The optimal number of factors aligns with the count of observed eigenvalues exceeding the 95th percentile (Longman et al., 1989) of the distribution of the associated randomly generated eigenvalues. Consequently, unidimensionality would be supported if only the first observed eigenvalue exceeds the corresponding randomly generated one.

The Minimum Average Partial (MAP) correlation statistic (Velicer, 1976) is derived from the average partial correlations among variables after sequentially eliminating the influence of factors, one at a time, in descending order of their eigenvalues. Following each stage, the squared average partial correlation among the items is calculated, and the number of factors that minimizes this value corresponds to the optimal solution. The first factor should produce the lowest squared average partial correlation to support unidimensionality.

References

(Buja & Eyuboglu, 1992) Buja, A., & Eyuboglu, N. (1992). Remarks on parallel analysis. *Multivariate Behavioral Research*, *27*(4), 509–540. https://doi.org/10.1207/s15327906mbr2704_2.

(Cattell, 1966) Cattell, R. B. (1966). The scree test for the number of factors. *Multivariate Behavioral Research*, *1*(2), 245–276. https://doi.org/10.1207/s15327906mbr0102_10.

(Horn, 1965) Horn, J. L. (1965). A rationale and test for the number of factors in factor analysis. *Psychometrika*, *30*(2), 179–185. https://doi.org/10.1007/BF02289447.

(Longman et al., 1989) Longman, R. S., Cota, A. A., Holden, R. R., & Fekken, G. C. (1989). A regression equation for the parallel analysis criterion in principal components analysis: Mean and 95th percentile eigenvalues. *Multivariate Behavioral Research*, *24*(1), 59–69. https://doi.org/10.1207/s15327906mbr2401_4.

Velicer (1976) Velicer, W. (1976). Determining the number of components from the matrix of partial correlations. *Psychometrika*, *41*(3), 321–327. https://doi.org/10.1007/BF02293557.

S2. Frequencies and percentages of score endorsement.

Item	Score				Missing
	0	1	2	3	
Total sample (n = 1912)					
1. Limit amount of food	980 (51.26%)	389 (20.35%)	295 (15.43%)	246 (12.87%)	2 (0.10%)
2. Long periods not eating	1609 (84.15%)	186 (9.73%)	69 (3.61%)	48 (2.51%)	0 (0.00%)
3. Thinking about food hinders concentration	1793 (93.78%)	83 (4.34%)	26 (1.36%)	10 (0.52%)	0 (0.00%)
4. Thinking about weight hinders concentration	1774 (92.78%)	96 (5.02%)	27 (1.41%)	15 (0.78%)	0 (0.00%)
5. Fear of gaining weight	1144 (59.83%)	442 (23.12%)	163 (8.53%)	163 (8.53%)	0 (0.00%)
6. Desire to lose weight	1203 (62.92%)	343 (17.94%)	172 (9.00%)	194 (10.15%)	0 (0.00%)
7. Vomiting or using laxatives	1888 (98.74%)	15 (0.78%)	6 (0.31%)	3 (0.16%)	0 (0.00%)
8. Exercising for controlling weight	1793 (93.78%)	79 (4.13%)	29 (1.52%)	11 (0.58%)	0 (0.00%)
9. Lost control over eating	1634 (85.46%)	201 (10.51%)	50 (2.62%)	27 (1.41%)	0 (0.00%)
10. Eating large amount of food	1664 (87.03%)	180 (9.41%)	50 (2.62%)	18 (0.94%)	0 (0.00%)
11. Weight/shape influenced self-concept	1141 (59.68%)	487 (25.47%)	205 (10.72%)	78 (4.08%)	1 (0.05%)
12. Dissatisfaction with weight/shape	737 (38.55%)	622 (32.53%)	382 (19.98%)	162 (8.47%)	9 (0.47%)
Women (n = 1170)					
1. Limit amount of food	575 (49.15%)	236 (20.17%)	188 (16.07%)	169 (14.44%)	2 (0.17%)
2. Long periods not eating	980 (83.76%)	119 (10.17%)	40 (3.42%)	31 (2.65%)	0 (0.00%)
3. Thinking about food hinders concentration	1091 (93.25%)	51 (4.36%)	21 (1.79%)	7 (0.60%)	0 (0.00%)
4. Thinking about weight hinders concentration	1079 (92.22%)	62 (5.30%)	17 (1.45%)	12 (1.03%)	0 (0.00%)
5. Fear of gaining weight	631 (53.93%)	292 (24.96%)	123 (10.51%)	124 (10.60%)	0 (0.00%)
6. Desire to lose weight	680 (58.12%)	229 (19.57%)	119 (10.17%)	142 (12.14%)	0 (0.00%)
7. Vomiting or using laxatives	1156 (98.80%)	9 (0.77%)	4 (0.34%)	1 (0.09%)	0 (0.00%)
8. Exercising for controlling weight	1108 (94.70%)	44 (3.76%)	16 (1.37%)	2 (0.17%)	0 (0.00%)
9. Lost control over eating	997 (85.21%)	123 (10.51%)	34 (2.91%)	16 (1.37%)	0 (0.00%)
10. Eating large amount of food	1015 (86.75%)	106 (9.06%)	37 (3.16%)	12 (1.03%)	0 (0.00%)
11. Weight/shape influenced self-concept	645 (55.13%)	321 (27.44%)	145 (12.39%)	58 (4.96%)	1 (0.09%)
12. Dissatisfaction with weight/shape	389 (33.25%)	400 (34.19%)	248 (21.20%)	126 (10.77%)	7 (0.60%)

cont.

	Score				
Item	0	1	2	3	Missing
Men (n = 742)					
1. Limit amount of food	405 (54.58%)	153 (20.62%)	107 (14.42%)	77 (10.38%)	0 (0.00%)
2. Long periods not eating	629 (84.77%)	67 (9.03%)	29 (3.91%)	17 (2.29%)	0 (0.00%)
3. Thinking about food hinders concentration	702 (94.61%)	32 (4.31%)	5 (0.67%)	3 (0.40%)	0 (0.00%)
4. Thinking about weight hinders concentration	695 (93.67%)	34 (4.58%)	10 (1.35%)	3 (0.40%)	0 (0.00%)
5. Fear of gaining weight	513 (69.14%)	150 (20.22%)	40 (5.39%)	39 (5.26%)	0 (0.00%)
6. Desire to lose weight	523 (70.49%)	114 (15.36%)	53 (7.14%)	52 (7.01%)	0 (0.00%)
7. Vomiting or using laxatives	732 (98.65%)	6 (0.81%)	2 (0.27%)	2 (0.27%)	0 (0.00%)
8. Exercising for controlling weight	685 (92.32%)	35 (4.72%)	13 (1.75%)	9 (1.21%)	0 (0.00%)
9. Lost control over eating	637 (85.85%)	78 (10.51%)	16 (2.16%)	11 (1.48%)	0 (0.00%)
10. Eating large amount of food	649 (87.47%)	74 (9.97%)	13 (1.75%)	6 (0.81%)	0 (0.00%)
11. Weight/shape influenced self-concept	496 (66.85%)	166 (22.37%)	60 (8.09%)	20 (2.70%)	0 (0.00%)
12. Dissatisfaction with weight/shape	348 (46.90%)	222 (29.92%)	134 (18.06%)	36 (4.85%)	2 (0.27%)

S3. Factor loading matrices.

	Total sample (n = 1912)			Women (n = 1170)			Men (n = 742)		
	Single factor	Two-factor		Single factor	Two-factor		Single factor	Two-factor	
		F1	F2		F1	F2		F1	F2
1. Limit amount of food	**0.68**	**0.85**	–0.18	**0.70**	**0.86**	–0.17	**0.66**	**0.81**	–0.16
2. Long periods not eating	**0.65**	**0.62**	0.04	**0.68**	**0.63**	0.06	**0.62**	**0.61**	0.01
3. Thinking about food hinders concentration	**0.83**	**0.64**	0.22	**0.83**	**0.76**	0.09	**0.82**	**0.44**	**0.41**
4. Thinking about weight hinders concentration	**0.85**	**0.60**	0.29	**0.84**	**0.69**	0.19	**0.86**	**0.48**	**0.42**
5. Fear of gaining weight	**0.91**	**0.92**	–0.01	**0.89**	**0.94**	–0.04	**0.93**	**0.84**	0.11
6. Desire to lose weight	**0.92**	**0.96**	–0.02	**0.91**	**0.92**	0.01	**0.93**	**0.99**	–0.04
7. Vomiting or using laxatives	**0.68**	**0.44**	0.27	**0.71**	**0.56**	0.18	**0.65**	0.20	**0.48**
8. Exercising for controlling weight	**0.64**	0.24	**0.46**	**0.65**	0.22	**0.50**	**0.69**	**0.40**	**0.33**
9. Lost control over eating	**0.82**	0.07	**0.88**	**0.81**	0.05	**0.89**	**0.86**	0.08	**0.89**
10. Eating large amount of food	**0.82**	–0.01	**0.97**	**0.81**	–0.01	**0.96**	**0.85**	–0.04	**0.99**
11. Weight/shape influenced self-concept	**0.77**	**0.52**	**0.31**	**0.76**	**0.44**	**0.39**	**0.78**	**0.54**	0.27
12. Dissatisfaction with weight/shape	**0.75**	**0.60**	0.18	**0.76**	**0.52**	0.29	**0.73**	**0.65**	0.10
Correlation with F2		0.75			0.74			0.78	

https://doi.org/10.3390/ejihpe16030037

S4. Parameter estimates.

Item	Discrimination	Threshold 1	Threshold 2	Threshold 3
Total sample (n = 1912)				
1. Limit amount of food	1.60 [1.44, 1.76]	−0.14 [−0.27, −0.01]	−1.40 [−1.55, −1.25]	−2.67 [−2.86, −2.48]
2. Long periods not eating	1.46 [1.26, 1.66]	−2.25 [−2.45, −2.05]	−3.51 [−3.77, −3.24]	−4.54 [−4.89, −4.18]
3. Thinking about food hinders concentration	2.52 [2.10, 2.93]	−4.65 [−5.21, −4.10]	−6.32 [−7.02, −5.61]	−7.90 [−8.86, −6.94]
4. Thinking about weight hinders concentration	2.75 [2.32, 3.19]	−4.68 [−5.24, −4.12]	−6.46 [−7.19, −5.74]	−7.89 [−8.83, −6.96]
5. Fear of gaining weight	3.62 [3.23, 4.00]	−1.10 [−1.35, −0.85]	−3.82 [−4.21, −3.44]	−5.44 [−5.92, −4.95]
6. Desire to lose weight	4.02 [3.56, 4.48]	−1.54 [−1.84, −1.25]	−3.81 [−4.23, −3.38]	−5.49 [−6.04, −4.95]
7. Vomiting or using laxatives	1.56 [1.04, 2.08]	−5.43 [−6.21, −4.66]	−6.46 [−7.41, −5.51]	−7.59 [−8.93, −6.25]
8. Exercising for controlling weight	1.42 [1.15, 1.68]	−3.46 [−3.78, −3.14]	−4.70 [−5.13, −4.27]	−6.06 [−6.72, −5.39]
9. Lost control over eating	2.48 [2.16, 2.80]	−3.18 [−3.51, −2.84]	−5.23 [−5.71, −4.75]	−6.69 [−7.32, −6.05]
10. Eating large amount of food	2.47 [2.14, 2.80]	−3.38 [−3.74, −3.02]	−5.37 [−5.87, −4.87]	−7.11 [−7.83, −6.40]
11. Weight/shape influenced self-concept	2.06 [1.86, 2.26]	−0.68 [−0.84, −0.52]	−2.77 [−2.99, −2.54]	−4.68 [−5.02, −4.34]
12. Dissatisfaction with weight/shape	1.94 [1.77, 2.12]	0.68 [0.53, 0.83]	−1.51 [−1.68, −1.34]	−3.53 [−3.78, −3.29]
Women (n = 1170)				
1. Limit amount of food	1.66 [1.45, 1.86]	−0.01 [−0.18, 0.16]	−1.26 [−1.44, −1.07]	−2.53 [−2.77, −2.29]
2. Long periods not eating	1.56 [1.29, 1.82]	−2.27 [−2.53, −2.00]	−3.60 [−3.95, −3.24]	−4.58 [−5.04, −4.12]
3. Thinking about food hinders concentration	2.56 [2.03, 3.09]	−4.57 [−5.25, −3.88]	−6.08 [−6.94, −5.22]	−7.90 [−9.11, −6.68]
4. Thinking about weight hinders concentration	2.68 [2.15, 3.21]	−4.46 [−5.12, −3.80]	−6.20 [−7.06, −5.33]	−7.47 [−8.55, −6.38]
5. Fear of gaining weight	3.33 [2.90, 3.76]	−0.45 [−0.73, −0.17]	−2.99 [−3.38, −2.60]	−4.63 [−5.15, −4.12]
6. Desire to lose weight	3.75 [3.22, 4.27]	−0.93 [−1.25, −0.61]	−3.11 [−3.56, −2.66]	−4.75 [−5.34, −4.17]
7. Vomiting or using laxatives	1.72 [1.02, 2.42]	−5.69 [−6.79, −4.60]	−6.79 [−8.12, −5.45]	−8.46 [−10.69, −6.23]
8. Exercising for controlling weight	1.45 [1.09, 1.81]	−3.70 [−4.15, −3.25]	−5.10 [−5.73, −4.47]	−7.37 [−8.83, −5.91]
9. Lost control over eating	2.35 [1.97, 2.73]	−3.03 [−3.43, −2.63]	−5.01 [−5.58, −4.44]	−6.57 [−7.36, −5.78]
10. Eating large amount of food	2.37 [1.97, 2.78]	−3.25 [−3.68, −2.81]	−5.03 [−5.62, −4.44]	−6.89 [−7.76, −6.02]
11. Weight/shape influenced self-concept	1.99 [1.74, 2.23]	−0.36 [−0.55, −0.17]	−2.42 [−2.68, −2.16]	−4.33 [−4.73, −3.93]
12. Dissatisfaction with weight/shape	1.97 [1.75, 2.20]	1.06 [0.86, 1.26]	−1.25 [−1.45, −1.05]	−3.20 [−3.49, −2.91]

cont.

Item	Discrimination	Threshold 1	Threshold 2	Threshold 3
Men (n = 742)				
1. Limit amount of food	1.48 [1.23, 1.72]	−0.33 [−0.53, −0.12]	−1.61 [−1.86, −1.36]	−2.90 [−3.22, −2.57]
2. Long periods not eating	1.34 [1.03, 1.66]	−2.24 [−2.56, −1.92]	−3.40 [−3.82, −2.98]	−4.52 [−5.09, −3.94]
3. Thinking about food hinders concentration	2.41 [1.72, 3.10]	−4.77 [−5.71, −3.83]	−6.80 [−8.05, −5.54]	−7.86 [−9.44, −6.28]
4. Thinking about weight hinders concentration	2.88 [2.10, 3.65]	−5.07 [−6.11, −4.04]	−6.97 [−8.30, −5.63]	−8.83 [−10.67, −7.00]
5. Fear of gaining weight	4.27 [3.45, 5.08]	−2.45 [−3.05, −1.85]	−5.79 [−6.79, −4.79]	−7.37 [−8.56, −6.18]
6. Desire to lose weight	4.35 [3.49, 5.21]	−2.65 [−3.29, −2.01]	−5.07 [−5.99, −4.14]	−6.81 [−7.97, −5.66]
7. Vomiting or using laxatives	1.44 [0.63, 2.26]	−5.21 [−6.36, −4.06]	−6.16 [−7.54, −4.77]	−6.86 [−8.56, −5.16]
8. Exercising for controlling weight	1.62 [1.17, 2.07]	−3.37 [−3.89, −2.85]	−4.48 [−5.13, −3.84]	−5.48 [−6.32, −4.64]
9. Lost control over eating	2.92 [2.30, 3.53]	−3.65 [−4.32, −2.98]	−5.95 [−6.89, −5.01]	−7.28 [−8.44, −6.11]
10. Eating large amount of food	2.78 [2.17, 3.40]	−3.78 [−4.47, −3.09]	−6.24 [−7.23, −5.24]	−7.75 [−9.07, −6.43]
11. Weight/shape influenced self-concept	2.11 [1.76, 2.46]	−1.21 [−1.50, −0.93]	−3.35 [−3.78, −2.93]	−5.30 [−5.95, −4.65]
12. Dissatisfaction with weight/shape	1.80 [1.52, 2.08]	0.12 [−0.11, 0.34]	−1.89 [−2.17, −1.61]	−4.14 [−4.61, −3.68]

https://doi.org/10.3390/ejihpe16030037

S5. Orlando and Thissen's (2003) $S\text{-}X^2$ item fit indices.

Item	$S\text{-}X^2$	df	RMSEA	*adj-p*
Total sample (n = 1912)				
1. Limit amount of food	56.63	45	0.012	0.172
2. Long periods not eating	103.99	59	0.020	0.001
3. Thinking about food hinders concentration	46.42	32	0.015	0.086
4. Thinking about weight hinders concentration	31.39	31	0.003	0.501
5. Fear of gaining weight	58.25	38	0.017	0.045
6. Desire to lose weight	76.58	34	0.026	<0.001
7. Vomiting or using laxatives	10.82	11	<0.001	0.501
8. Exercising for controlling weight	36.46	39	<0.001	0.586
9. Lost control over eating	69.43	46	0.016	0.043
10. Eating large amount of food	50.96	36	0.015	0.086
11. Weight/shape influenced self-concept	56.24	48	0.010	0.258
12. Dissatisfaction with weight/shape	84.85	43	0.023	0.001
Women (n = 1170)				
1. Limit amount of food	39.95	44	<0.001	0.646
2. Long periods not eating	77.74	51	0.021	0.039
3. Thinking about food hinders concentration	42.91	28	0.021	0.085
4. Thinking about weight hinders concentration	25.35	28	<0.001	0.646
5. Fear of gaining weight	62.31	33	0.028	0.018
6. Desire to lose weight	53.54	32	0.024	0.039
7. Vomiting or using laxatives	7.22	5	0.020	0.273
8. Exercising for controlling weight	23.52	20	0.012	0.317
9. Lost control over eating	43.53	30	0.020	0.105
10. Eating large amount of food	41.12	32	0.016	0.220
11. Weight/shape influenced self-concept	54.97	45	0.014	0.220
12. Dissatisfaction with weight/shape	60.30	40	0.021	0.062

cont.

https://doi.org/10.3390/ejihpe16030037

Item	*S-X*^{2}	df	RMSEA	*adj-p*
Men (n = 742)				
1. Limit amount of food	63.77	39	0.029	0.030
2. Long periods not eating	57.22	37	0.027	0.054
3. Thinking about food hinders concentration	15.05	13	0.015	0.365
4. Thinking about weight hinders concentration	41.68	13	0.055	0.001
5. Fear of gaining weight	29.15	22	0.021	0.241
6. Desire to lose weight	21.12	25	<0.001	0.686
7. Vomiting or using laxatives	4.84	4	0.017	0.365
8. Exercising for controlling weight	26.55	25	0.009	0.413
9. Lost control over eating	32.39	19	0.031	0.068
10. Eating large amount of food	31.31	20	0.028	0.102
11. Weight/shape influenced self-concept	35.47	30	0.016	0.339
12. Dissatisfaction with weight/shape	59.92	33	0.033	0.017

References

Orlando, M., & Thissen, D. (2003). Further investigation of the performance of S-X^2: An item fit index for use with dichotomous item response theory models. *Applied Psychological Measurement*, *27*(4), 289–298. https://doi.org/10.1177/0146621603027004004.

S6. Slope modification indices for the metric invariance model.

Item	X^2	df	*p*	*adj-p*
1. Limit amount of food	2.135	1	0.144	0.529
2. Long periods not eating	1.500	1	0.221	0.529
3. Thinking about food hinders concentration	0.259	1	0.611	0.705
4. Thinking about weight hinders concentration	0.109	1	0.741	0.741
5. Fear of gaining weight	4.018	1	0.045	0.529
6. Desire to lose weight	1.193	1	0.275	0.549
7. Vomiting or using laxatives	0.357	1	0.550	0.705
8. Exercising for controlling weight	0.210	1	0.647	0.705
9. Lost control over eating	2.141	1	0.143	0.529
10. Eating large amount of food	0.726	1	0.394	0.676
11. Weight/shape influenced self-concept	0.268	1	0.605	0.705
12. Dissatisfaction with weight/shape	1.788	1	0.181	0.529

S7. Details of the correlational analyses.

S7.1 Correlations with age as a metric variable.

Women: $r = 0.103$ [0.046, 0.160], $t(1168) = 3.557$, $p < 0.001$.
Men: $r = 0.018$ [–0.054, 0.090], $t(740) = 0.497$, $p = 0.620$.

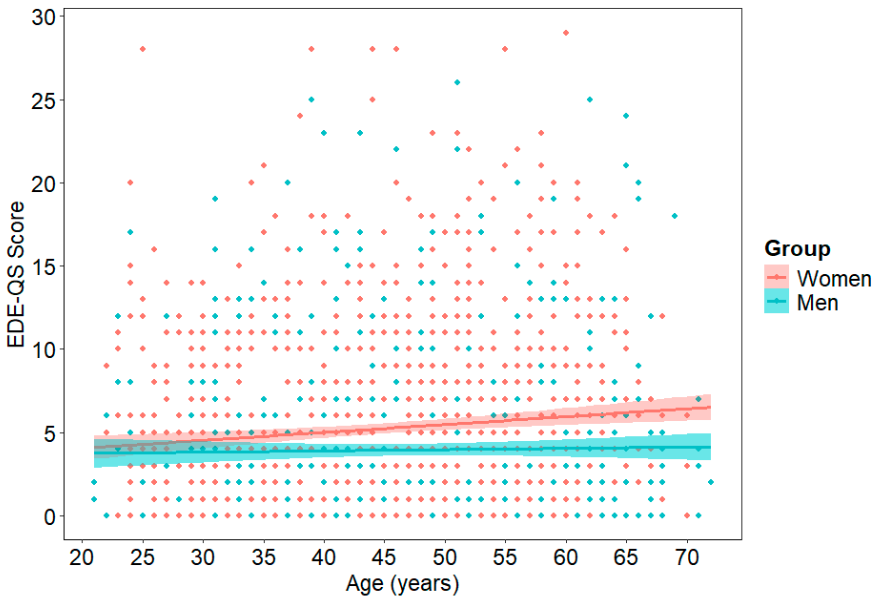

S7.2 Correlations with age as a categorical variable.

Women: $F(2, 1167) = 8.786$, $p < 0.001$, $\eta_p^2 = 0.015$ [0.003, 0.031].

Age group	n	M	SD
Young (age < 45)	574	4.564	4.945
Middle (45 ≤ age < 65)	565	5.894	5.779
Elder (age ≥ 65)	31	5.355	4.484

Note: n: sample size; M = mean; SD = standard deviation.

Contrast	Estimate	SE	df	t	FDR–adjusted p–value	d
Young–Middle	–1.329	0.317	1167	–4.190	<0.001	0.245 [0.130, 0.361]
Young–Elder	–0.790	0.987	1167	–0.801	0.585	0.047 [–0.068, 0.162]
Middle–Elder	0.539	0.988	1167	0.546	0.585	0.032 [–0.083, 0.147]

Note: SE: standard error; df = degrees of freedom; t: t statistic; FDR = false discovery rate; d: Cohen's effect size.

Men: $F(2, 739) = 1.338$, $p = 0.263$, $\eta^2 = 0.004$ [0.000, 0.015].

Age group	n	M	SD
Young (age < 45)	317	3.962	4.768
Middle (45 ≤ age < 65)	378	3.820	4.711
Elder (age ≥ 65)	47	5.043	6.083

Note: n: sample size; M = mean; SD = standard deviation.

Contrast	Estimate	SE	df	t	FDR–adjusted p–value	d
Young–Middle	0.142	0.368	739	0.386	0.700	0.028 [–0.116, 0.173]
Young–Elder	–1.080	0.755	739	–1.431	0.229	0.105 [–0.039, 0.250]
Middle–Elder	–1.222	0.747	739	–1.636	0.229	0.120 [–0.024, 0.265]

Note: SE: standard error; df = degrees of freedom; t: t statistic; FDR = false discovery rate; d: Cohen's effect size.

S7.3 Correlations with Body Mass Index (BMI) as a categorical variable.

Women: $r = 0.387$ [0.331, 0.441], $t(917) = 12.723$, $p < 0.001$.
Men: $r = 0.328$ [0.257, 0.395], $t(635) = 8.745$, $p < 0.001$.

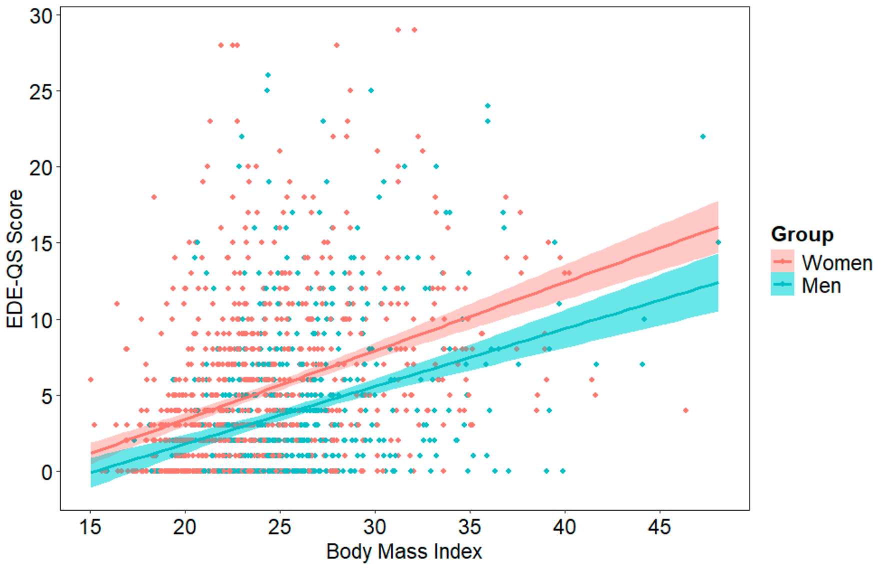

S7.4 Correlations with Body Mass Index (BMI) as a categorical variable.

Women: $F(3, 915) = 46.160$, $p < 0.001$, $\eta^2 = 0.131$ [0.092, 0.171].

BMI group	n	M	SD
Underweight	52	2.462	3.578
Normal weight	531	3.991	4.689
Overweight	227	6.700	5.445
Obese	109	9.211	5.825

Note: n: sample size; M = mean; SD = standard deviation.

Contrast	Estimate	SE	df	t	FDR–adjusted p–value	d
Under–Normal	–1.529	0.723	915	–2.114	0.035	0.140 [0.010, 0.270]
Under–Over	–4.239	0.765	915	–5.539	<0.001	0.366 [0.234, 0.498]
Under–Obese	–6.749	0.839	915	–8.045	<0.001	0.532 [0.398, 0.666]
Normal–Over	–2.710	0.395	915	–6.865	<0.001	0.454 [0.321, 0.587]
Normal–Obese	–5.220	0.523	915	–9.973	<0.001	0.659 [0.523, 0.796]
Over–Obese	–2.511	0.580	915	–4.328	<0.001	0.286 [0.155, 0.417]

Note: SE: standard error; df = degrees of freedom; t: t statistic; FDR = false discovery rate; d: Cohen's effect size.

Men: $F(3, 633) = 18.693$, $p < 0.001$, $\eta^2 = 0.081$ [0.043, 0.122].

BMI group	n	M	SD
Underweight	6	1.000	1.265
Normal weight	258	2.961	4.220
Overweight	278	4.047	4.355
Obese	95	7.042	6.390

Note: n: sample size; M = mean; SD = standard deviation.

Contrast	Estimate	SE	df	t	FDR–adjusted p–value	d
Under–Normal	–1.961	1.920	633	–1.022	0.307	0.081 [–0.075, 0.237]
Under–Over	–3.047	1.918	633	–1.588	0.135	0.126 [–0.030, 0.283]
Under–Obese	–6.042	1.957	633	–3.088	0.004	0.245 [0.088, 0.403]
Normal–Over	–1.086	0.402	633	–2.701	0.011	0.215 [0.058, 0.372]
Normal–Obese	–4.081	0.558	633	–7.315	0.000	0.582 [0.419, 0.744]
Over–Obese	–2.995	0.552	633	–5.422	0.000	0.431 [0.272, 0.591]

Note: SE: standard error; df = degrees of freedom; t: t statistic; FDR = false discovery rate; d: Cohen's effect size.

S7.5 Correlations with depression.

Women: $r = 0.377$ [0.326, 0.425], $t(1162) = 13.864$, $p < 0.001$.
Men: $r = 0.351$ [0.286, 0.413], $t(737) = 10.172$, $p < 0.001$.

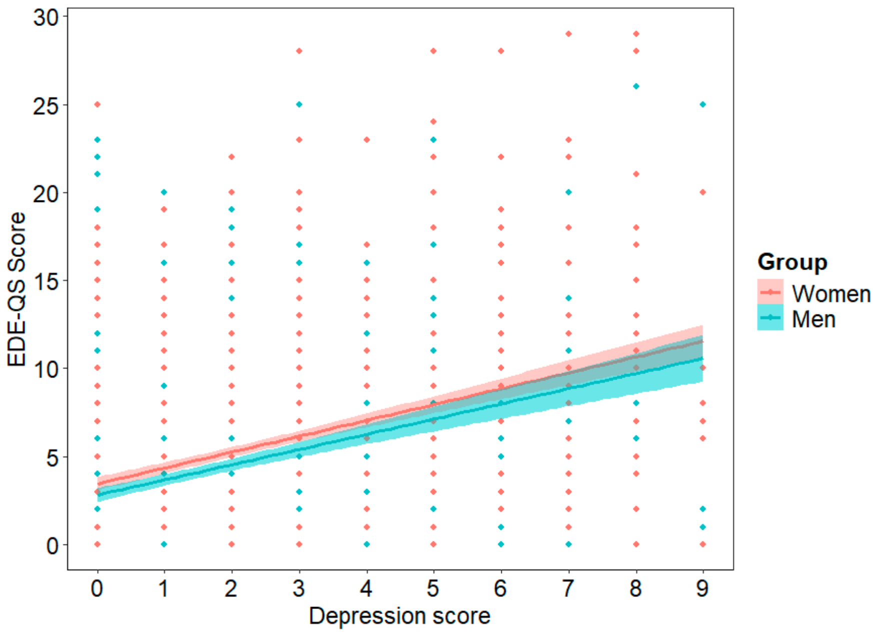

S7.6 Correlations with anxiety.

Women: $r = 0.380$ [0.330, 0.428], $t(1162) = 13.997$, $p < 0.001$.
Men: $r = 0.318$ [0.252, 0.381], $t(737) = 9.108$, $p < 0.001$.

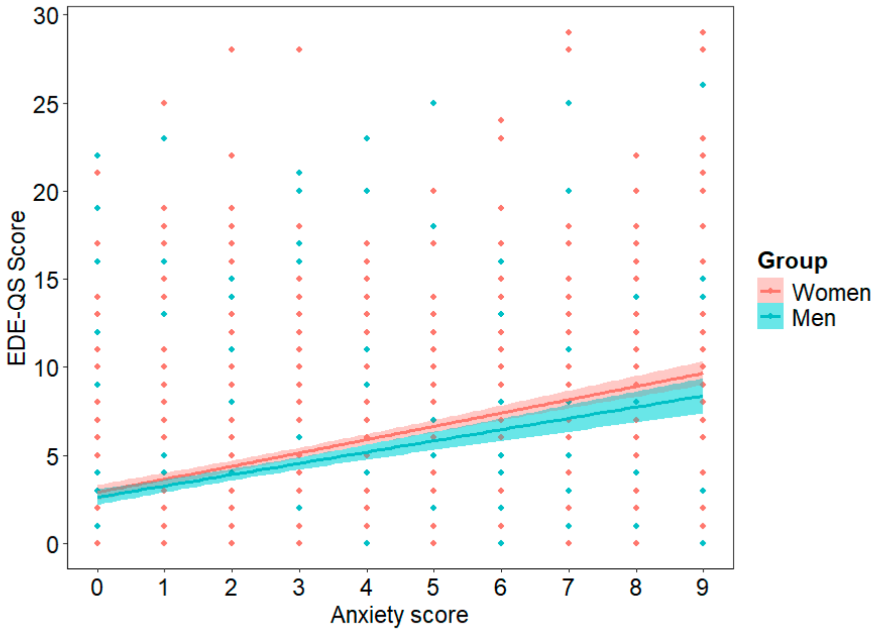

S7.7 Correlations with happiness.

Women: $r = –0.290$ [–0.342, –0.237], $t(1168) = –10.373$, $p < 0.001$.
Men: $r = –0.259$ [–0.325, –0.190], $t(739) = –7.278$, $p < 0.001$.

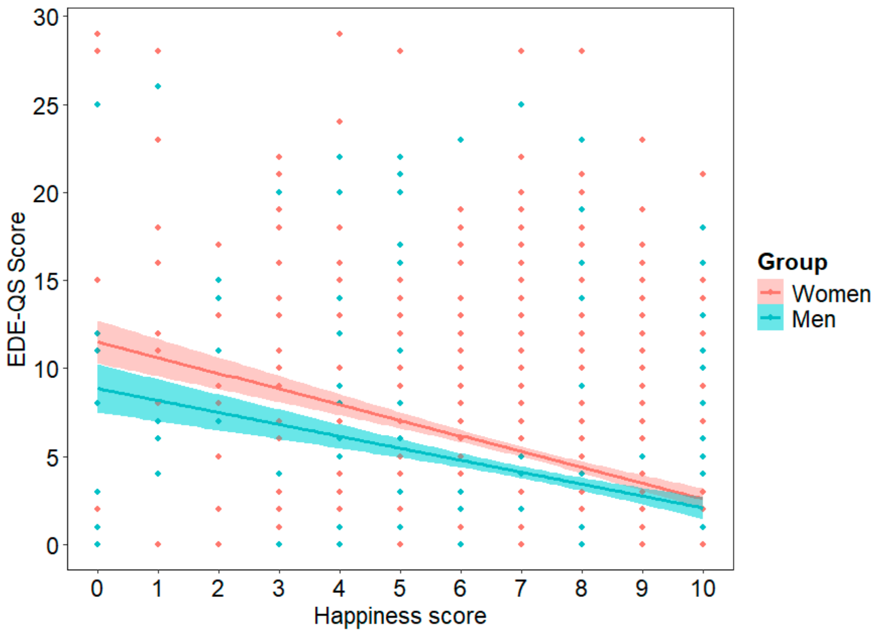

S7.8 Correlations with effort.

Women: $r = 0.155$ [0.098, 0.211], $t(1154) = 5.329$, $p < 0.001$.
Men: $r = 0.161$ [0.090, 0.231], $t(732) = 4.418$, $p < 0.001$.

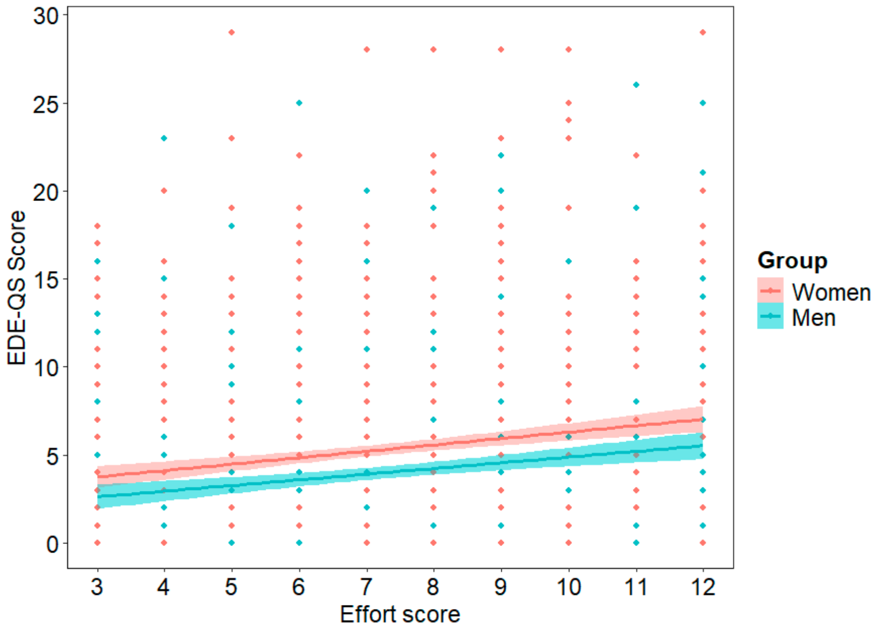

S7.9 Correlations with reward.

Women: r = –0.203 [–0.258, –0.147], t(1154) = –7.054, $p < 0.001$.
Men: r = –0.189 [–0.258, –0.119], t(732) = –5.219, $p < 0.001$.

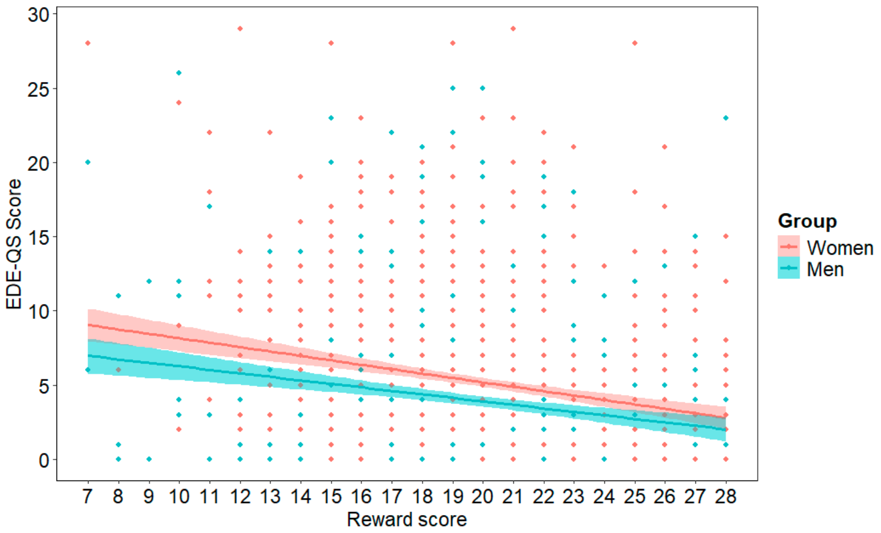

S7.10 Correlations with Effort/Reward Imbalance Index (ERI).

Women: r = 0.215 [0.159, 0.269], t(1154) = 7.463, $p < 0.001$.
Men: r = 0.209 [0.139, 0.277], t(732) = 5.777, $p < 0.001$.

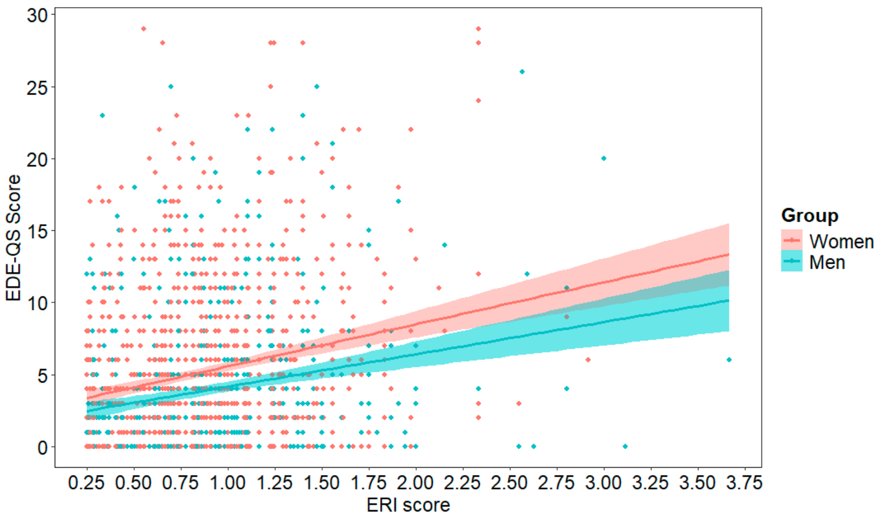

S7.11 Correlations with sleep quality.

Women: $r = 0.349$ [0.297, 0.398], $t(1161) = 12.679$, $p < 0.001$.
Men: $r = 0.316$ [0.249, 0.379], $t(733) = 9.011$, $p < 0.001$.

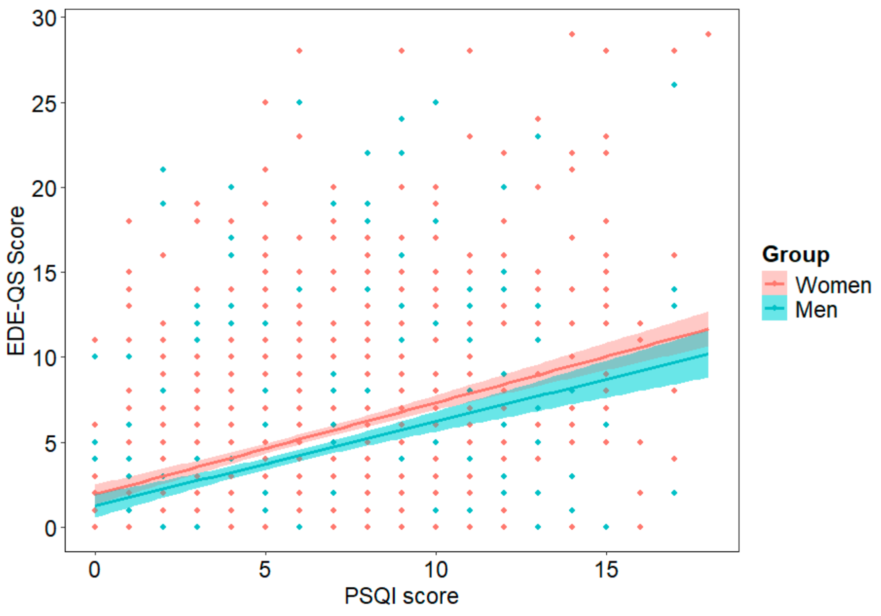

S7.12 Correlations with health literacy.

Women: $r = -0.155$ [–0.210, –0.098], $t(1160) = -5.327$, $p < 0.001$.
Men: $r = -0.211$ [–0.279, –0.141], $t(733) = -5.865$, $p < 0.001$.

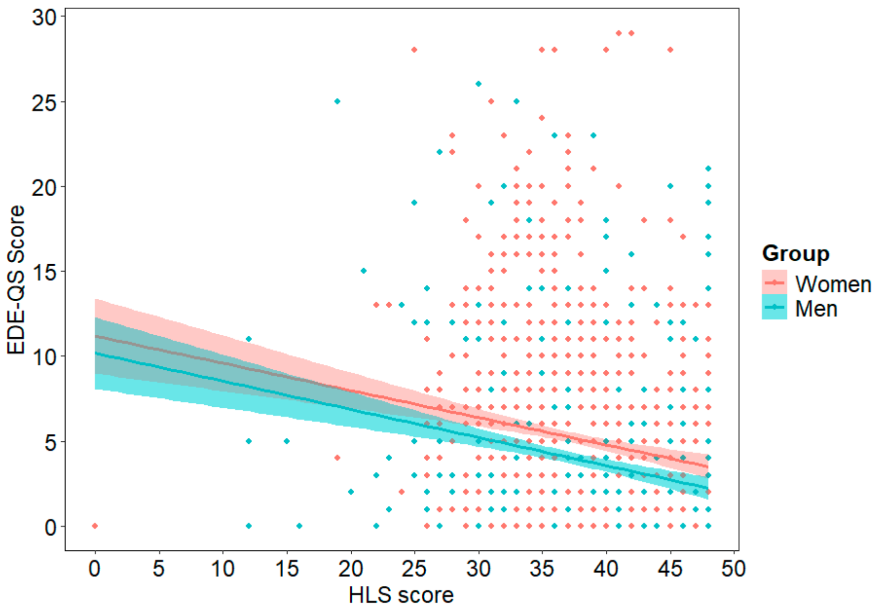

S7.13 Correlations with healthy diet literacy.

Women: r = –0.038 [–0.095, 0.020], t(1148) = –1.281, p = 0.200.
Men: r = –0.120 [–0.191, –0.049], t(733) = –3.293, p = 0.001.

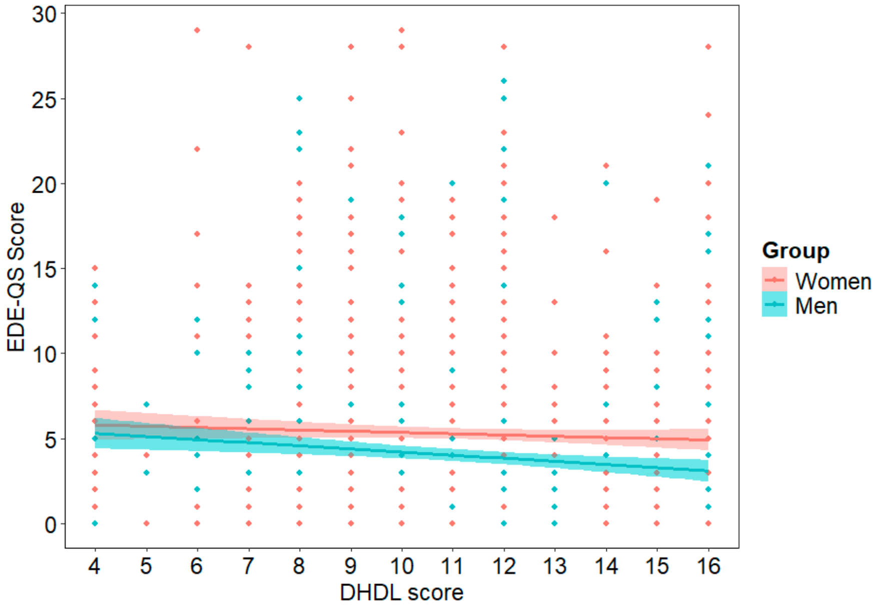

S7.13 Correlations with night work.

Women: t(591.48) = 0.270, p = 0.787, d = 0.02 [–0.11, 0.14].

Night work	n	M	SD
No	856	5.252	5.482
Yes	314	5.159	5.136

Note: n: sample size; M = mean; SD = standard deviation.

Men: t(365.84) = 2.255, p = 0.025, d = 0.18 [0.02, 0.34].

Night work	n	M	SD
No	557	4.171	4.997
Yes	185	3.319	4.254

Note: n: sample size; M = mean; SD = standard deviation.

S7.14 Correlations with violence.

Women: $r = 0.123$ [0.066, 0.179], $t(1168) = 4.232$, $p < 0.001$.
Men: $r = 0.143$ [0.072, 0.213], $t(740) = –3.937$, $p < 0.001$.

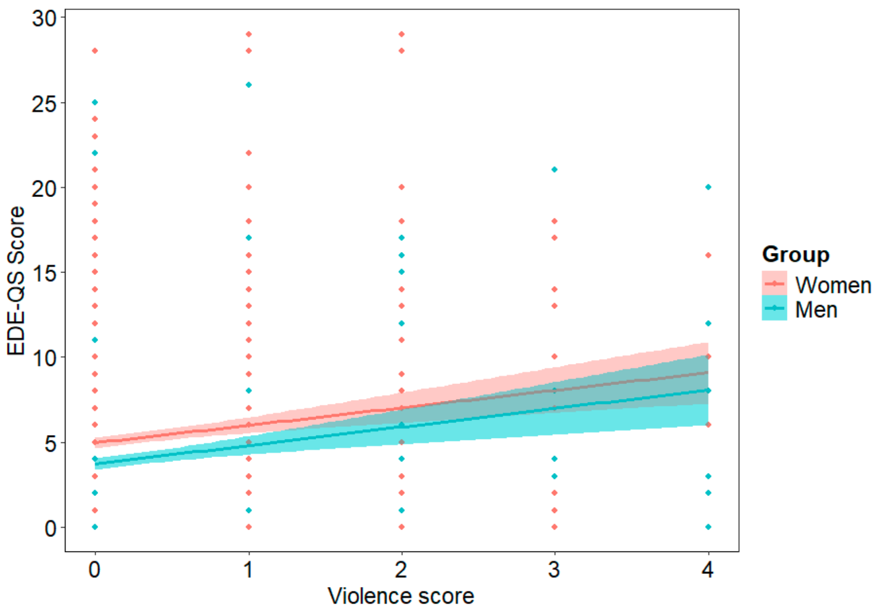

S7.15 Correlations with smoking.

Women: $F(3, 1165) = 0.119$, $p = 0.949$, $\eta^2 < 0.001$ [0.000, 0.001].

Smoking	n	M	SD
Never smoked	560	5.193	5.500
Quit smoking	201	5.428	5.025
Sometimes	135	5.126	5.694
Always	273	5.194	5.298

Note: n: sample size; M = mean; SD = standard deviation.

Contrast	Estimate	SE	df	t	FDR–adjusted p–value	d
Never–Quit	–0.235	0.444	1165	–0.530	0.997	0.031 [–0.084, 0.146]
Never–Sometimes	0.067	0.518	1165	0.129	0.997	0.008 [–0.107, 0.122]
Never–Always	–0.001	0.398	1165	–0.003	0.997	0.000 [–0.115, 0.115]
Quit–Sometimes	0.302	0.601	1165	0.503	0.997	0.029 [–0.085, 0.144]
Quit–Always	0.234	0.502	1165	0.466	0.997	0.027 [–0.088, 0.142]
Sometimes–Always	–0.068	0.568	1165	–0.120	0.997	0.007 [–0.108, 0.122]

Note: SE: standard error; df = degrees of freedom; t: t statistic; FDR = false discovery rate; d: Cohen's effect size.

Men: $F(3, 737) = 2.252$, $p = 0.081$, $\eta^2 = 0.009$ [0.000, 0.024].

Smoking	n	M	SD
Never smoked	321	3.720	4.506
Quitted smoking	175	4.234	5.298
Sometimes	86	5.047	5.083
Always	159	3.572	4.750

Note: n: sample size; M = mean; SD = standard deviation.

Contrast	Estimate	SE	df	t	FDR–adjusted p–value	d
Never–Quit	–0.515	0.453	737	–1.136	0.308	0.084 [–0.061, 0.228]
Never–Sometimes	–1.327	0.586	737	–2.266	0.071	0.167 [0.022, 0.312]
Never–Always	0.147	0.468	737	0.315	0.753	0.023 [–0.121, 0.168]
Quitted–Sometimes	–0.812	0.635	737	–1.279	0.308	0.094 [–0.050, 0.239]
Quitted–Always	0.662	0.528	737	1.253	0.308	0.092 [–0.052, 0.237]
Sometimes–Always	1.474	0.646	737	2.284	0.071	0.168 [0.023, 0.313]

Note: SE: standard error; df = degrees of freedom; t: t statistic; FDR = false discovery rate; d: Cohen's effect size.

S7.16 Correlations with drinking.

Women: 0.048 [–0.021, 0.117], $t(1168) = 1.364$, $p = 0.172$.
Men: –0.016 [–0.099, 0.066], $t(738) = –0.384$, $p = 0.701$.

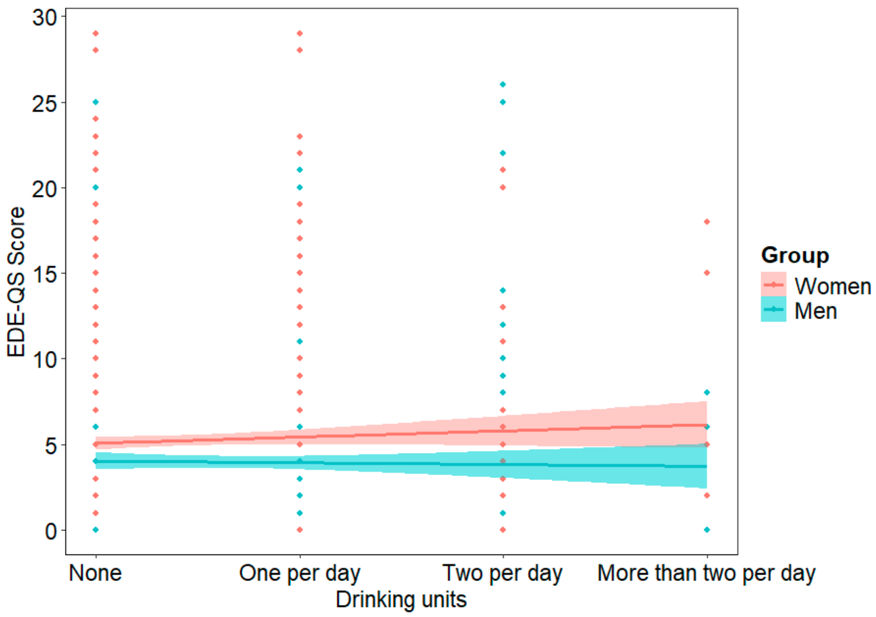

Disclaimer/Publisher's Note: The statements, opinions and data contained in all publications are solely those of the individual author(s) and contributor(s) and not of MDPI and/or the editor(s). MDPI and/or the editor(s) disclaim responsibility for any injury to people or property resulting from any ideas, methods, instructions or products referred to in the content.
